# A solvable model for strongly interacting nonequilibrium excitons

**DOI:** 10.1073/pnas.2424663122

**Published:** 2025-03-14

**Authors:** Zhenhao Song, Tessa Cookmeyer, Leon Balents

**Affiliations:** ^a^Department of Physics, University of California, Santa Barbara, CA 93106-4030; ^b^Kavli Institute for Theoretical Physics, University of California, Santa Barbara, CA 93106-4030; ^c^Canadian Institute for Advanced Research, Toronto, ON M5G 1M1, Canada; ^d^French American Center for Theoretical Science, CNRS, Kavli Institute for Theoretical Physics, Santa Barbara, CA 93106-4030

**Keywords:** Bose–Hubbard model, exciton, nonequilibrium, Lindblad master equation

## Abstract

We study the driven-dissipative Bose-Hubbard model with an all-to-all hopping term in the system Hamiltonian, while subject to incoherent pumping and decay from the environment. This system is naturally probed in several recent experiments on excitons in WS_2_/WSe_2_ moiré systems, as well as quantum simulators. By positing a particular form of coupling to the environment, we derive the Lindblad jump operators and show that, in certain limits, the system admits a closed-form expression for the steady-state density matrix. Away from the exactly solvable regions, the steady state can be obtained numerically for 100s to 1,000s of sites. We study the nonequilibrium phase diagram and phase transitions, which qualitatively matches the equilibrium phase diagram, agreeing with the intuition that increasing the intensity of the light is equivalent to changing the bosonic chemical potential. However, the steady states are far from thermal states, and the nature of the phase transitions is changed.

## Significance

Excitons, bound states of electrons and holes, can be formed by shining a laser on a semiconductor. Recent experiments have suggested that sufficiently intense laser excitation applied to a WS_2_-WSe_2_ bilayer creates a “Mott insulator” of excitons in which they fill a lattice and can no longer easily move around. Since the excitons have a finite lifetime before decaying, this phase transition is fundamentally “nonequilibrium.” By developing a solvable toy model that goes beyond the standard approach of the literature, we theoretically study and characterize a nonequilibrium phase transition out of the Mott insulator phase and validate some of the experimental heuristics. The results provide a relevant exemplar of a phase transition in an open quantum system.

The Bose–Hubbard model is the paradigmatic model of interacting bosons on a lattice and describes a transition between a Mott insulating and superfluid phase (1). There are now several platforms for experimentally realizing this model such as trapped bosonic atoms (2–6) or photon modes (7–9). In the latter case, interactions are induced through circuit quantum electrodynamics (QED) (10, 11), cavity QED (12–15), semiconductor platforms (16–18), or Rydberg atoms (19, 20), and a lattice can be produced by interconnecting these platforms (15, 18, 21). Excitons in transition-metal dichalcogenide (TMD) moiré bilayers (22–24) or artificial lattices (25) provide another emerging solid-state realization of Bose–Hubbard physics, where the interactions are naturally implemented through strong dipole repulsions. All these experimental settings move beyond the study of equilibrium physics as these systems either controllably couple to their environments or have finite lifetimes for the bosons (10, 26).

We are most interested in understanding several recent experiments potentially realizing bosonic Mott physics with excitons in WSe_2_/WS_2_ moiré bilayers (27–31). The experiments see a finite energy shift in the photoluminescence spectrum interpreted as an on-site exciton–exciton interaction with multiple excitons per site. There is considerable evidence that the excitons enter an interaction-dominated Mott-insulating regime at sufficiently large intensities of light (27, 30). The inherently nonequilibrium nature of these experiments, given the necessity of continuously pumping the system full of decaying excitons, naturally raises the question of how much these reported nonequilibrium Mott phases resemble their equilibrium counterpart, and whether an analog of the superfluid phase exists out of equilibrium.

The Bose–Hubbard model,

[1] $$\begin{array}{c} H_{0}=\mu \sum_{i}n_{i}+\frac{U}{2}\sum_{i}n_{i}\left(n_{i}-1\right)-\sum_{i,j}t_{i,j}b_{i}^{†}b_{j}^{†}, \end{array}$$

subject to dissipation and driving, is the prototypical model for studying strongly interacting open quantum systems. Here, $b_{i}^{†}\left(b_{i}^{†}\right)$ denotes the bosonic annihilation (creation) operator at site i, and $n_{i}=b_{i}^{†}b_{i}^{†}$ is the boson number operator there. The parameters U, $t_{\mathrm{ij}}$, and μ are the on-site Coulomb repulsion, hopping amplitude between sites i and j, and chemical potential, respectively. For moiré excitons, μ is simply the excitation gap of a single exciton in the minimum of the moiré potential.

The drive is often coherently added to the total Hamiltonian, $H_{\;\text{tot}}=H_{0}+Fb_{i}^{†}+F^{*}b_{i}$, with F being the Rabi frequency associated to a coherent driving field, e.g. electromagnetic wave mode of a cavity, and the decay is implemented through a Lindblad master equation for the density matrix ρ (32–34),

[2] $$\begin{array}{c} \dot{\rho }=-i\left[H_{0},\rho \right]+\sum_{k,k^{\prime }}\gamma_{k,k^{\prime }}L\left(L_{k},L_{k^{\prime }}^{†}\right)\left[\rho \right], \end{array}$$

where $L\left(A,B\right)\left[\rho \left(t\right)\right]=A\rho \left(t\right)B-\frac{1}{2}\left\{BA,\rho \left(t\right)\right\}$, $L_{k}$ are the jump operators and $\gamma_{k,k^{\prime }}$ are the associated damping rates. By including suitable Lindblad jump operators, we can include incoherent driving as the reverse process of decay. In our work, we assume all driving is incoherent and set F=0.

This system has been numerically studied with numerous methods: mean-field theory (MFT) (35–41), cluster MFT (42, 43), the positive P representation (37, 44), the corner-space renormalization group method (45), functional renormalization group using a Keldysh path integral (32, 46), quantum trajectories (38, 43, 47), matrix-product-state-based methods (48–50), and a truncated hierarchy of correlators (51). These studies all assume a simple and decoupled form of Lindblad jump operators, i.e. $L_{i,-}=b_{i}$, which works well for describing nearly isolated noninteracting atoms. In general, however, such an assumption does not hold for a strongly correlated system when $\gamma_{k,k^{\prime }}/U\ll 1$.

In this work, we emphasize that the explicit form of jump operators is determined by the system Hamiltonian itself, as well as the coupling with the environment, which can significantly modify the Lindblad master equation and the properties of the steady state. In order to fully understand the phases and dynamics in these nonequilibrium systems, it is useful to have prototypical solvable models (52). Here, we study the Bose Hubbard Hamiltonian Eq. **1** with an all-to-all hopping for N sites, i.e. $t_{\mathrm{ij}}=t/N$ for any i,j, which may be considered a sort of infinite-dimensional limit. The all-to-all hopping mimics the effect of a mean-field analysis at the equilibrium level and allows us to move beyond mean-field with analytical control. Remarkably, in such an approach we are able to derive the explicit form of the Lindblad jump operators, analytically obtain closed-form solutions of the steady state in certain limits, and numerically solve the system for up to thousands of sites in others.

**Fig. 1.**
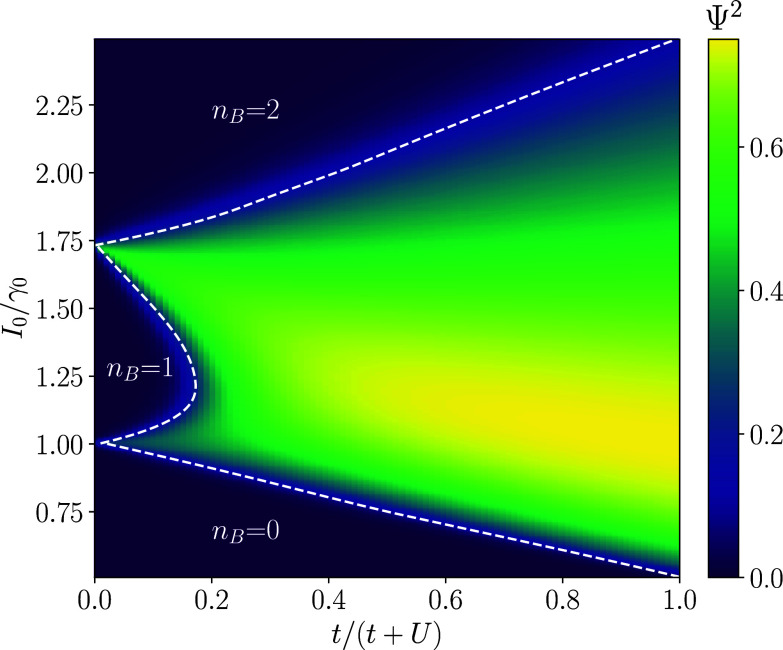
We plot the order parameter $\Psi^{2}=\sum_{i,j}\left\langle b_{i}^{†}b_{j}\right\rangle /N^{2}$ computed for the steady-state density matrix $\rho_{\mathrm{ss}}$, extrapolated to the N→∞ limit as a function of the ratio of exciton production and decay rates, $I_{0}/\gamma_{0}$, and t/(t+U). $I_{0}\left(\gamma_{0}\right)$ are pumping (decay) rates at energy ω=μ. Damping rates at other energies are specified as in Section 4. The phase boundaries are estimated using the entropy per site $S=-\;\text{Tr}[\rho_{\mathrm{ss}}\ln\left(\rho_{\mathrm{ss}}\right)]/N$, which is maximal at the phase transition. We see that $n_{B}=0,1,2$ in the lobes as indicated on the plot in *SI Appendix*.

Our main results are summarized in the phase diagram, Fig. 1, a color map of the superfluid order parameter $\Psi^{2}=\sum_{i,j}\left\langle b_{i}^{†}b_{j}\right\rangle /N^{2}$, as a function of the ratio of exciton production and decay rates $I_{0}/\gamma_{0}$ (appearing as damping rates in the Lindblad equation Eq. **2**) and t/(t+U). $I_{0}$ and $\gamma_{0}$ are pumping and decay rates at energy ω=μ, the detailed structure of which are specified in Section 4. Similar to the equilibrium model, we see that there are two phases: Mott-insulator phases with an integer values of $n_{B}=\left(1/N\right)\sum_{i}\left\langle b_{i}^{†}b_{i}\right\rangle$ and a superfluid phase identified as a nonzero value of $\Psi^{2}=\left(1/N^{2}\right)\sum_{i,j}\left\langle b_{i}^{†}b_{j}\right\rangle$ with a generic value of $n_{B}$. Although the steady-state density matrices are not of a (thermal) equilibrium form, the nonequilibrium and equilibrium phase diagrams resemble each other lending credence to the qualitative understanding of the experiment as tuning the boson chemical potential. We additionally study the critical properties of the phase transition. Notably, the Mott–Mott phase transition at t=0 (either from $n_{B}=0$ to $n_{B}=1$, or $n_{B}=1$ to $n_{B}=2$), which is simply a level crossing at the equilibrium level, becomes a continuous phase transition and the critical exponents for the Mott–superfluid transition are distinct from their equilibrium values.

## 1. Derivation of the Master Equation

We will analyze the Bose–Hubbard Hamiltonian, Eq. **1** with all-to-all hopping between the N sites, $t_{i,j}=t/N$, and in the presence of coupling to the environment. We will assume that μ≫U,t as is relevant for the physical system.

We now proceed to derive the Lindblad master equation, Eq. **2**, in the weak-coupling limit for this model. We follow the standard procedure (53, 54): First, we start with a general coupling form between the system and environment $H_{I}=\sum_{\alpha }S_{\alpha }⊗R_{\alpha }$, where $S_{\alpha }$ ($R_{\alpha }$) are Hermitian operators that act on the system (environment), and the index α labels different operators. Then, we decompose $S_{\alpha }=\sum_{j}S_{\alpha ;j}$ by eigenoperators of the system Hamiltonian $H_{0}$, which are defined by the relation $\left[H_{0},S_{\alpha ;j}\right]=-\omega_{j}S_{\alpha ;j}$. The Lindblad jump operators are nothing but the eigenoperators $S_{\alpha ;j}$, and the damping rate $\gamma_{\alpha ;j}$ is given by $\int_{-\infty }^{+\infty }dse^{i\omega_{j}s}\left\langle R_{\alpha }^{†}\left(s\right)R_{\alpha }\left(0\right)\right\rangle$. With these operators in hand, the equation of motion for the system’s density matrix can be written out explicitly according to Eq. **2**.

Since the excitons emit light when they decay, we know that they interact with the quantized electromagnetic field. A typical dipole interaction takes the form $-\sum_{i}{\vec{D}}_{i}\cdot {\vec{E}}_{i}$, with ${\vec{D}}_{i}∼\left(b_{i}^{†}+b_{i}^{†}\right)$. In momentum space, after the rotating-wave approximation, it can be expressed as (55)

[3] $$\begin{array}{c} \sum_{k,\pm }g_{k,\pm }a_{k,\pm }b_{k}^{†}\approx \frac{1}{\sqrt{N}}\sum_{k,\pm }g_{k,\pm }a_{k,\pm }\sum_{i}b_{i}^{†}, \end{array}$$

where $g_{k,\pm }$ is related to the polarization of the light mode. We used $k\cdot r_{i}\ll 1$ since the relevant wavelength of light (≈700 nm) is much larger than the distance between moiré unit cells (≈7.5 nm) (56). Therefore, as opposed to the common assumption where each site couples to different modes in the bath, we assume the environment couples with the system in a collective way, reminiscent of the Dicke model (57). Note that $g_{k}∼O\left(\sqrt{N/V}\right)$ where V is the volume available to the photon modes, and we suppress the light’s polarization for simplicity. With such an assumption, we may write the coupling as

[4a] $$\begin{array}{cc} H_{I} & =B^{†}R^{†}+R^{†}B^{†} \end{array}$$

with


[4b] $$\begin{array}{cc} B & \equiv \sum_{i}b_{i}, \end{array}$$

where R denotes the environment operator. Though we only argued above for the decay of excitons to have such a form, treating the production and decay on equal footing allows us to make theoretical progress.

We do not specify the explicit forms of R since they could be complicated in general. Specifically, the long-lived interlayer excitons which form the exciton lattice are generated through at least a two-step process, where intralayer excitons are photoexcited and then converted to interlayer excitons through some relaxation process (23). We therefore consider only the incoherent contributions of $H_{I}$ to the exciton system.

We now decompose B in $H_{I}$ in terms of eigenoperators, which, in general, requires diagonalizing $H_{0}$. Note that the coupling to the environment and the Hamiltonian itself is invariant under all permutations of the sites; we also assume the system’s initial state (before turning on exciton-producing effects) is the vacuum ρ=|0⟩⟨0| since μ>0, which has permutation symmetry as well. Therefore, the only states that are accessed are in the fully symmetric sector of this permutation symmetry and it is sufficient to diagonalize $H_{0}$ in this sector.

Similar to the procedure adopted in multilevel systems (58), we parameterize the sector with the normalized states

[5] $$\begin{array}{c} |\vec{n}\rangle =\frac{1}{\sqrt{N!\prod_{i}\left(n_{i}\right)!}}\sum_{\sigma \in S_{N}}\sigma \left|\left(n_{0},n_{1},...,n_{M}\right)\right\rangle , \end{array}$$

where $\vec{n}$ is a vector in (M+1) dimensional space, $S_{N}$ is the symmetric group, σ is the permutation operator that shuffles site i to σ(i), and the state $\left|(n_{0},n_{1},...,n_{M})\right\rangle$ has the first $n_{0}$ sites being unoccupied, the next $n_{1}$ sites having one boson occupied, etc. We have chosen a maximum number of allowed bosons per site, M, and $\sum_{i}n_{i}=N$.

It is convenient to define transition operators $O_{n,m}\equiv \sum_{i}\left(|n\right\rangle {\left\langle m|\right)}_{i}⊗\prod_{j\neq i}1_{j}$ which changes site i from having m bosons to n bosons and acts as the identity on all other sites. Note that $O_{n,m}\sigma =\sigma O_{n,m}$ for all $\sigma \in S_{N}$. We can then find

[6] $$\begin{array}{c} O_{n,m}|\vec{n}\rangle =|\vec{n}-{\hat{e}}_{m}+{\hat{e}}_{n}\rangle \left\{\begin{array}{cc} \sqrt{n_{m}\left(n_{n}+1\right)} & \;\text{if}\;n\neq m\\ n_{m} & \;\text{if}\;n=m \end{array}\right., \end{array}$$

where ${\hat{e}}_{i}$ are the unit vectors and $n_{m}$ are the components of $\vec{n}$.

Letting ${\hat{N}}_{B}$ be the total boson number operator with eigenvalues $N_{B}$, we use these relations to write $H_{0}$ in the $|\vec{n}\rangle$ basis:

[7] $$\begin{array}{cc} H_{0}\left|\vec{n}\right\rangle & =\left(\mu N_{B}+\frac{U}{2}\sum_{n}n\left(n-1\right)n_{n}\right)\left|\vec{n}\right\rangle \\ & \;-\frac{t}{N}\sum_{\mathrm{nm}}\sqrt{nmn_{m}n_{m-1}^{\left(m\right)}n_{n-1}^{\left(m\right)}\left(n_{n}^{\left(m\right)}+1\right)}\left|{\vec{n}}^{\left(m\right)}-{\hat{e}}_{n-1}+{\hat{e}}_{n}\right\rangle , \end{array}$$

where ${\vec{n}}^{\left(m\right)}=\vec{n}-{\hat{e}}_{m}+{\hat{e}}_{m-1}$. For a given M and N, we can numerically find the eigenbasis within each $N_{B}$ sector where only the ratio t/U determines the eigenvectors. We denote the eigenvectors $|\alpha ,N_{B}\rangle$ where each sector has a different number of allowed α.

We now eigen-decompose the coupling operator $B=\sum_{i}b_{i}$ within the fully permutation-symmetric sector to find

$$\begin{array}{c} B=\sum_{\lambda }B_{\lambda }=\sum_{\left(\alpha ,\beta ,N_{B}\right)}B_{\left(\alpha ,\beta ,N_{B}\right)}|\alpha ,N_{B}-1\rangle \langle \beta N_{B}|. \end{array}$$

where $B_{\left(\alpha ,\beta ,N_{B}\right)}=\langle \alpha ,N_{B}-1|B|\beta N_{B}\rangle$ and $\lambda =(\alpha ,\beta ,N_{B})$ is a combined index. Note that the operators in the sum have energy $\left[H,|\alpha ,N_{B}-1\right\rangle \left\langle \beta N_{B}|\right]=-\left(E_{\beta ,N_{B}}-E_{\alpha ,N_{B}-1}\right)|\alpha ,N_{B}-1\rangle \langle \beta N_{B}|=-\omega_{\alpha ,\beta ,N_{B}}|\alpha ,N_{B}-1\rangle \langle \beta N_{B}|$.

With the eigenoperators in hand, we can immediately write out the interaction-picture Lindblad equation (ignoring the Lamb shift)

[8] $$\begin{array}{cc} \dot{\rho }\left(t\right) & =\sum_{\lambda ,\lambda^{\prime }:\omega_{\lambda }=\omega_{\lambda^{\prime }}}\gamma \left(\omega_{\lambda }\right)L\left(B_{\lambda }^{†}\left(\omega_{\lambda }\right),B_{\lambda^{\prime }}^{†}\left(\omega_{\lambda^{\prime }}\right)\right)\left[\rho \left(t\right)\right]\\ & \;+\sum_{\lambda ,\lambda^{\prime }:\omega_{\lambda }=\omega_{\lambda^{\prime }}}I\left(\omega_{\lambda }\right)L\left(B_{\lambda }^{†}\left(\omega_{\lambda }\right),B_{\lambda^{\prime }}^{†}\left(\omega_{\lambda^{\prime }}\right)\right)\left[\rho (t)\right]. \end{array}$$

The first (second) line corresponds to decay (pumping) processes. Equality between $\omega_{\lambda }$ and $\omega_{\lambda^{\prime }}$ is determined by the condition $|\omega_{\lambda }-\omega_{\lambda }^{\prime }|\ll \tau^{-1}$, where τ sets the relaxation timescale of the system. The specifics of the environment enter only through

[9] $$\begin{array}{cc} \gamma (\omega ) & =\int_{-\infty }^{\infty }dse^{i\omega s}\left\langle R\left(s\right)R^{†}\right\rangle \\ I(\omega ) & =\int_{-\infty }^{\infty }dse^{-i\omega s}\left\langle R{\left(s\right)}^{†}R\right\rangle \end{array}$$

and we have assumed $\left\langle R\left(s\right)R\right\rangle =\left\langle R^{†}\left(s\right)R^{†}\right\rangle =0$ (and $\tau^{-1}$ is set by γ(ω),I(ω) which are assumed to be the smallest energy scales in the weak-coupling limit).

We now write out Eq. **8** in the eigenbasis, $|\alpha ,N_{B}\rangle$. One difficulty in solving the resulting equation is the presence of coherences (i.e. off-diagonal terms of the density matrix in the basis of energy eigenstates). As we derive in *SI Appendix*, the Lindblad master equation can only generate coherences within energy-degenerate sectors, and therefore, if there is no degeneracy, the steady-state density matrix is diagonal. However, there is a weaker condition that ensures that the $B_{\lambda }\left(\omega_{\lambda }\right)$ operators never generate coherences when acting on a diagonal ρ (see *SI Appendix*): $\omega_{\alpha ,\beta ,N_{B}}=\omega_{\alpha^{\prime },\beta ,N_{B}}$ ($\omega_{\alpha ,\beta ,N_{B}}=\omega_{\alpha ,\beta^{\prime },N_{B}}$) only when $B_{\left(\alpha ,\beta ,N_{B}\right)}=0$ or $B_{\left(\alpha^{\prime },\beta ,N_{B}\right)}=0$ ($B_{\left(\alpha ,\beta ,N_{B}\right)}=0$ or $B_{\left(\alpha ,\beta^{\prime },N_{B}\right)}=0$), respectively. In our analytic or numerical results, we always check that the above condition is satisfied for this system.

We can therefore write $\rho =\sum_{\alpha ,N_{B}}\rho_{\alpha ,N_{B}}|\alpha ,N_{B}\rangle \langle \alpha ,N_{B}|$ and

[10] $$\begin{array}{cc} {\dot{\rho }}_{a,N_{B}} & =\sum_{\lambda =(\alpha ,a,N_{B})}{\left|B_{\lambda }\right|}^{2}\left(I\left(\omega_{\lambda }\right)\rho_{\alpha ,N_{B}-1}-\gamma \left(\omega_{\lambda }\right)\rho_{a,N_{B}}\right)\\ & \;+\sum_{\lambda =(a,\beta ,N_{B}+1)}{\left|B_{\lambda }\right|}^{2}\left(\gamma \left(\omega_{\lambda }\right)\rho_{\beta ,N_{B}+1}-I\left(\omega_{\lambda }\right)\rho_{a,N_{B}}\right). \end{array}$$

As a check on our derivation, we can set I(ω) and γ(ω) to represent a thermal bath of free bosons. In this case, the Kubo-Martin-Schwinger condition holds allowing us to write $\gamma \left(\omega \right)=A\left(\omega \right)(1+n_{B}\left(\omega /T\right))$ and $I\left(\omega \right)=A\left(\omega \right)n_{B}\left(\omega /T\right)$ where A(ω) is a function only of ω and $n_{B}\left(x\right)={\left(e^{x}-1\right)}^{-1}$ is the Bose–Einstein distribution. The function A(ω) is determined by the specifics of the environment [e.g. $A\left(\omega \right)∼\omega^{3}$ for the photon field (54)]. We numerically find that the steady-state density matrix, ${\dot{\rho }}_{a,N_{B}}=0$ has the thermal form $\rho_{a,N_{B}}∼e^{-E_{a,N_{B}}/T}$. In general, we consider nonzero I(ω) as resulting from light-induced, rather than thermal, production of excitons.

## 2. Key Observables, Phases, and Exponents

Once the steady state satisfying ${\dot{\rho }}_{a,N_{B}}=0$ from Eq. **10** is determined, there are two key observables to evaluate

[11] $$\begin{array}{c} n_{B}=\frac{1}{N}\sum_{i}\left\langle b_{i}^{†}b_{i}\right\rangle ,\;\Psi^{2}=\frac{1}{N^{2}}\sum_{i,j}\left\langle b_{i}^{†}b_{j}\right\rangle =\frac{\left\langle B^{†}B\right\rangle }{N^{2}}. \end{array}$$

The two phases present in our model are Mott-insulating phases with integer exciton density $n_{B}\in \left\{0,1,2,...\right\}$ (and $\Psi^{2}=0$) and a superfluid phase with generic $n_{B}$ and $\Psi^{2}\neq 0$. In the equilibrium case, the superfluid phase is determined by the nonzero expectation $\left\langle b_{i}\right\rangle$ (or equivalently the long-range order of $\left\langle b_{j}^{†}b_{i}\right\rangle$), and $\Psi^{2}$ is our analogous observable.

In addition to the phases themselves, we study the phase transitions and extract the critical exponents. We will tune across the phase transitions primarily by adjusting the ratio r(ω)=I(ω)/γ(ω) of the exciton production and decay strengths. With μ≫U,t, we may Taylor expand r(ω) around ω=μ and the transition occurs when $r\left(\mu \right)=r_{1}$ reaches a critical value $r_{c}$.

At a continuous (second-order) phase transition, there is an order parameter χ that takes a particular form close to the phase transition

[12] $$\begin{array}{c} \chi =\left\{\begin{array}{cc} \chi_{0}{\left(r_{1}-r_{c}\right)}^{\beta } & r_{1}>r_{c}\\ 0 & r_{1}<r_{c} \end{array}\right., \end{array}$$

where $\chi_{0}$ is a constant, and the correlation length ξ of the system will diverge as $\xi ∼{\left(r_{1}-r_{c}\right)}^{-\nu }$. The exponents β and ν are universal numbers set by the phase transition itself, independent of the exact microscopic model. For systems of a finite length L, this diverging correlation length is cut off and will lead to corrections of the critical behavior that depend on ξ/L. Replacing the correlation length by ${\left(r_{1}-r_{c}\right)}^{-\nu }$ leads to the finite-size scaling form for $\chi =L^{-\beta /\nu }f_{\chi }\left(L^{1/\nu }\left(r_{1}-r_{c}\right)\right)$, where $f_{\chi }\left(x\right)$ is a finite-size scaling function (see e.g. ref. 59).

The all-to-all hopping in our system makes it difficult to define a correlation length, so we define the exponent λ as the analog for ν in our system. At the Mott–Mott transition from the $n_{B}=0$ to $n_{B}=1$ Mott phases, the order parameter is simply $n_{B}$, which therefore has the form

[13] $$\begin{array}{c} n_{B}=N^{-\beta_{m}/\lambda_{m}}f_{n_{B},m}\left(N^{1/\lambda_{m}}\left(r_{1}-r_{c}\right)\right), \end{array}$$

where $\beta_{m}$, $\lambda_{m}$ are the critical exponents and $f_{n_{B},m}\left(x\right)$ is the scaling function for $n_{B}$ at the Mott–Mott transition. Similarly, at the Mott–superfluid (Mott–Mott) transition, the order parameter is $\Psi^{2}$ and takes the scaling form

[14] $$\begin{array}{c} \Psi^{2}=N^{-\beta_{s}/\lambda_{s}}f_{\Psi^{2},s}\left(N^{1/\lambda_{s}}\left(r_{1}-r_{c}\right)\right) \end{array}$$

with similarly defined $\beta_{s}$, $\lambda_{s}$ and $f_{\Psi^{2},s}\left(x\right)$. Note that the exponents $\lambda_{m/s}$ and $\beta_{m/s}$ and scaling functions do not depend on the exact definition of the tuning parameter used to move between the two phases, and they can distinguish between different critical points.

## 3. Analytic Results

Due to needing to find the eigenvectors of $H_{0}$, Eq. **7**, we cannot make additional analytic progress in most cases. We will treat the general problem numerically in the next section, but there are two cases where more analytic progress can be made.^*^

### 3.1. *t* = 0.

In this limit, the eigenvectors of $H_{0}$ are simply the $|\vec{n}\rangle$ states defined above. Instead of simplifying Eq. **10**), we note that

[15] $$\begin{array}{c} B=\sum_{j=1}^{M}\sqrt{j}O_{j-1,j} \end{array}$$

and $\left[H,O_{j-1,j}\right]=-\left[\mu +U\left(j-1\right)\right]O_{j-1,j}=-\omega_{j}O_{j-1,j}$. We can therefore write out the equivalent of Eq. **8**

[16] $$\begin{array}{cc} \dot{\rho }\left(t\right) & =\sum_{j}\gamma \left(\omega_{j}\right)jL\left(O_{j-1,j},O_{j-1,j}^{†}\right)\left[\rho \left(t\right)\right]\\ & \;+\sum_{j}I\left(\omega_{j}\right)jL\left(O_{j-1,j}^{†},O_{j-1,j}\right)\left[\rho \left(t\right)\right]. \end{array}$$

We now substitute $\rho =\sum_{\vec{n},\vec{m}}\rho_{\vec{n},\vec{m}}|\vec{n}\rangle \langle \vec{m}|$. Using Eq. **6** and the fact that we start from a vacuum state, one can check that the condition $\rho_{\vec{n},\vec{m}}=0$ when $\vec{n}\neq \vec{m}$, is preserved under time evolution. Therefore, we may instead write $\rho \left(t\right)=\sum_{\vec{n}}\rho_{\vec{n}}\left(t\right)|\vec{n}\rangle \langle \vec{n}|$.

We can explicitly write out the steady-state equation as

[17] $$\begin{array}{cc} 0 & =\sum_{j=1}^{M}jn_{j-1}\left(n_{j}+1\right)\left(\gamma \left(\omega_{j}\right)\rho_{\vec{n}-{\hat{e}}_{j-1}+{\hat{e}}_{j}}-I\left(\omega_{j}\right)\rho_{\vec{n}}\right)\\ & \;+\sum_{j=1}^{M}jn_{j}\left(n_{j-1}+1\right)\left(I\left(\omega_{j}\right)\rho_{\vec{n}-{\hat{e}}_{j}+{\hat{e}}_{j-1}}-\gamma \left(\omega_{j}\right)\rho_{\vec{n}}\right), \end{array}$$

where the above equation holds for all $\vec{n}$ and $n_{j}$ denote components of $\vec{n}$. A sufficient “detailed-balance”-like condition for the steady state is that all terms in the sum are zero,

[18] $$\begin{array}{c} \gamma \left(\omega_{j}\right)\rho_{\vec{n}-{\hat{e}}_{j-1}+{\hat{e}}_{j}}=I\left(\omega_{j}\right)\rho_{\vec{n}}, \end{array}$$

which remarkably specifies the solution in this case. (In general, the equivalent set of equations will not hold as they overdetermine the solution.) It can easily be checked that the steady state is given explicitly as

[19] $$\begin{array}{cc} \rho_{\vec{n}} & =\rho_{0}\prod_{i=1}^{M}{\left(\prod_{j=1}^{i}\frac{I(\omega_{j})}{\gamma (\omega_{j})}\right)}^{n_{i}}=\rho_{0}\prod_{i=1}^{M}{\left(r(\omega_{i})\right)}^{\sum_{j=i}^{M}n_{j}}, \end{array}$$

where we have defined $r\left(\omega_{j}\right)\equiv I\left(\omega_{j}\right)/\gamma \left(\omega_{j}\right)=r_{j}$. This form of the steady state manifestly reveals that we can set the maximum number of bosons per site we need to consider, M, by the condition $r(\omega_{M})\ll 1$, when t=0.

We now analyze the case M=2 in detail, which can be mapped to the case of a three-level system with collective decay and pumping, and thus can be potentially implemented in cavity experiments. We can rewrite the density matrix as $\rho_{\vec{n}}=\rho_{0}r_{1}^{n_{1}}{\left(r_{1}r_{2}\right)}^{n_{1}+n_{2}}$ (and we assume that $r_{j}>r_{j+1}$). We can compute the average number of bosons per site $n_{B}=\left\langle N_{B}\right\rangle /N$ as

[20] $$\begin{array}{cc} n_{B} & =\left\{\begin{array}{cc} 0+\frac{1}{N}\frac{r_{1}+\left(2-3r_{1}\right)r_{1}r_{2}}{\left(r_{1}-1\right)\left(r_{1}r_{2}-1\right)}+... & \;\text{if}r_{1}^{N},r_{2}^{N}\ll 1\\ 1+\frac{1}{N}\frac{1-r_{1}r_{2}}{\left(r_{1}-1\right)\left(r_{2}-1\right)}+... & \;\text{if}r_{1}^{-N},r_{2}^{N}\ll 1\\ 2+\frac{1}{N}\frac{3-2r_{2}-r_{1}r_{2}}{\left(r_{2}-1\right)\left(r_{1}r_{2}-1\right)}+... & \;\text{if}r_{1}^{N},r_{2}^{N}\gg 1. \end{array}\right. \end{array}$$

We can see, in the thermodynamic limit, that our model has a sharp transition from an $n_{B}=0$ to $n_{B}=1$ to $n_{B}=2$ state as we increase the ratio $r_{1}$ and $r_{2}$. We identify these regions as Mott insulators (and we can numerically check that $\Psi^{2}\to 0$). We can analyze these critical points by evaluating

[21] $$\begin{array}{c} n_{B}=\left\{\begin{array}{cc} \frac{1}{2}+\frac{1}{12}\left(r_{1}-1\right)N+... & \;\text{if}r_{1}\approx 1,r_{2}^{N}\ll 1\\ \frac{3}{2}+\frac{1}{12}\left(r_{2}-1\right)N+... & \;\text{if}r_{2}\approx 1,r_{1}^{N}\gg 1 \end{array}\right. \end{array}$$

implying that $n_{B}$ has the scaling form $n_{B}=N^{-\beta_{m}/\lambda_{m}}f_{n_{B},m}\left(N^{1/\lambda_{m}}\left(r_{j}-1\right)\right)$ with $\lambda_{m}=1$ and $\beta_{m}=0$. We expect that the values of $\lambda_{m},\beta_{m}$ are insensitive to increasing M.

In equilibrium, this transition is first order since it is simply a level crossing of the two competing ground states, $|N{\hat{e}}_{j}\rangle$ to $|N{\hat{e}}_{j+1}\rangle$. However, in the nonequilibrium setting, as we approach the phase transition there are more and more states in the Hilbert space that contribute to the steady-state density matrix, which allows for a continuous phase transition to emerge between $\rho_{\mathrm{ss}}\approx |N{\hat{e}}_{j}\rangle \langle N{\hat{e}}_{j}|$ to $\rho_{\mathrm{ss}}\approx |N{\hat{e}}_{j+1}\rangle \langle N{\hat{e}}_{j+1}|$.

### 3.2. *M* = 1.

If we consider the case of hardcore bosons, i.e. U→∞, we can make additional progress as well because there is only one state per (fully symmetrized) sector, $N_{B}$, and therefore the eigenstates of $H_{0}$ can be specified as $|N_{B}\rangle$. The eigenenergy of the states is given by

[22] $$\begin{array}{c} E_{N_{B}}=\mu N_{B}-\frac{t}{N}N_{B}\left(N-N_{B}+1\right) \end{array}$$

and we can evaluate

[23] $$\begin{array}{c} B=\sum_{N_{B}=1}^{N}\sqrt{N_{B}\left(N-N_{B}+1\right)}|N_{B}-1\rangle \langle N_{B}| \end{array}$$

so $B_{\lambda }=B_{N_{B}}=\sqrt{N_{B}\left(N-N_{B}+1\right)}$ and these have energies $\omega_{N_{B}}=E_{N_{B}}-E_{N_{B}-1}=\mu -t\left(1-2\left(N_{B}-1\right)/N\right)$. When t is nonzero, we see that the energy will take on a range of values from μ−|t|<ω<μ+|t| in the thermodynamic limit.

Since all $\omega_{N_{B}}$ are unique, we still have no coherences and can write down the density matrix equation

[24] $$\begin{array}{cc} {\dot{\rho }}_{m} & =m\left(N-m+1\right)(I\left(\omega_{m}\right)\rho_{m-1}-\gamma \left(\omega_{m}\right)\rho_{m})\\ & \;+\left(m+1\right)\left(N-m\right)(\gamma \left(\omega_{m+1}\right)\rho_{m+1}-I\left(\omega_{m+1}\right)\rho_{m}), \end{array}$$

where m is specifying $N_{B}$. Again noting that a sufficient condition for the steady-state solution is

[25] $$\begin{array}{c} \gamma \left(\omega_{m}\right)\rho_{m}=I\left(\omega_{m}\right)\rho_{m-1} \end{array}$$

we find

[26] $$\begin{array}{c} \rho_{m}=\rho_{0}\prod_{j=1}^{m}\frac{I(\omega_{j})}{\gamma (\omega_{j})}=\rho_{0}\prod_{j=1}^{m}r\left(\omega_{j}\right) \end{array}$$

If t=0, we see that $\omega_{m}=\mu$ for all m, so we quickly derive Eq. **19** for M=1.

We will now show that, in addition to the $n_{B}=0$ and $n_{B}=1$ Mott phases seen before, there is an intermediate phase. We first assume the pump-to-decay ratio $r\left(\omega_{j}\right)=r_{j}$ decreases monotonically with ω, which fits the intuition that higher energy excitations are prone to decay more quickly. Defining $r_{<}\equiv r\left(\mu -t\right)$ [$r_{>}\equiv r\left(\mu +t\right)$], we can recover the Mott phases from before by computing $\rho_{m}=\rho_{0}r_{<}^{m}e^{\left(r_{<}^{\prime }tm^{2}\right)/\left(r_{<}N\right)}$$\left[\rho_{N-m}=\rho_{N}r_{>}^{-m}e^{\left(r_{>}^{\prime }tm^{2}\right)/\left(r_{>}N\right)}\right]$ when $r_{<}\ll 1$ ($r_{>}\gg 1$), respectively, where $r_{</>}^{\prime }$ are the corresponding values of $r^{\prime }\left(\omega \right)=dr\left(\omega \right)/d\omega <0$ (see *SI Appendix* for additional calculation details for this section).

Next, we consider the case $r_{<}>1>r_{>}$. There then exists some index $j^{*}$ where $r_{j^{*}}=1$. From Eqs. **25** and **26**, we can find as N→∞

[27] $$\begin{array}{c} \rho_{j^{*}\pm m}\approx \rho_{j^{*}}e^{-\delta m^{2}}, \end{array}$$

where $\delta =-r_{j^{*}}^{\prime }t/N>0$ from the monotonic decrease assumption.

We can deduce this density matrix represents an additional phase by measuring $\Psi^{2}$. Since M=1, we see that $B^{†}B|N_{B}\rangle =N_{B}\left(N-N_{B}+1\right)\left|N_{B}\right\rangle$. In the three aforementioned limits, we find

[28] $$\begin{array}{c} \Psi^{2}=\left\{\begin{array}{cc} \frac{r_{<}}{N}{\left(1-r_{<}\right)}^{-1}+... & \;\text{if}r_{<}\ll 1\\ \frac{j^{*}}{N}\left(1-\frac{j^{*}}{N}\right)+... & \;\text{if}r_{j^{*}}=1\;\text{and}j^{*}∼O\left(N\right)\\ \frac{1}{N}{\left(1-r_{>}^{-1}\right)}^{-1}+... & \;\text{if}r_{>}\gg 1 \end{array}\right. \end{array}$$

As above, we can observe a critical scaling emerge as $r_{<}\to 1$. Using Eq. **26**, we can find

[29] $$\begin{array}{c} \Psi^{2}\approx \frac{1}{N^{1/2}}\frac{\int_{0}^{\infty }\;dxx\;\exp\left[\frac{r_{<}^{\prime }t}{r_{<}}x^{2}-\left(1-r_{<}\right)\sqrt{N}x\right]}{\int_{0}^{\infty }\;dx\;\exp\left[\frac{r_{<}^{\prime }t}{r_{<}}x^{2}-\left(1-r_{<}\right)\sqrt{N}x\right]}. \end{array}$$

which has the scaling form $\Psi^{2}=N^{-1/2}f_{\Psi^{2},s}\left(N^{1/2}\left(r_{<}-1\right)\right)$, implying that $\lambda_{s}=2$ and $\beta_{s}=1$. A similar finite-size scaling form, with the same value of $\lambda_{s}$, can be derived for $n_{B}$.

We note that the M=1 hardcore boson case can actually be mapped to an Ising model, with corresponding interactions and dissipations. Various cases have been studied (60–62), but with a single jump operator which corresponds to cavity loss or cavity pumping. When the Lindblad jump operators are derived from the interacting Hamiltonian, the physics becomes more distinct.

## 4. Numerical Results

From the analytic results, we can see there are two phases distinguished by $\Psi^{2}$. If $\Psi^{2}\to 0$, then we are in a Mott insulating phase where $n_{B}$ is approximately an integer. Otherwise, $n_{B}$ takes an intermediate value, and $\Psi^{2}$ is nonzero, like in the superfluid phase. By solving the system numerically, we can explore what happens when t≠0 and more than one boson per site is allowed.

In order to proceed numerically, we must specify I(ω) and γ(ω). We take $\gamma \left(\omega \right)=\gamma_{0}{\left(\omega /\mu \right)}^{3}$, the correct form for photon-driven decay, and we assume $I\left(\omega \right)=I_{0}$ for simplicity.^†^ For the moiré TMD system (27), the realistic excitation gap μ≈1.5 eV and the repulsion U≈30 meV. However, we fix μ=150 meV as μ only affects $\gamma^{\prime }\left(\omega \right)$ [and consequently $r^{\prime }\left(\omega \right)$] which we need to be large to achieve numerical convergence. We let (t+U) set the energy scale, and vary both t/(t+U) and $r\left(\mu \right)=I_{0}/\gamma_{0}$ to map out the phase diagram and study the critical exponents.

Once t≠0 and M≥2, we can only numerically determine the Lindblad operators and energy eigenstates, and the equivalent conditions to Eqs. **18** and **25** cannot all be satisfied. Instead, we construct the Liouvillian superoperator L satisfying $\dot{\rho }=L\rho$ from Eq. **10** and search for the steady-state ρ that has eigenvalue zero. We can numerically evaluate the M=2 case up to a large system size of N∼1,000. In this case, when t=0, the various $N_{B}$ sectors are nondegenerate implying that the steady-state density matrix is diagonal, as we argued above. We check numerically that this nondegeneracy persists when t≠0.

In addition to measuring $n_{B}$ and $\Psi^{2}$, we measure the entropy S=−Tr[ρln(ρ)] and the Liouvillian gap, $\Delta_{L}$ determined from the smallest real part of any of the nonzero eigenvalues of L (63). These two quantities allow us to better distinguish between the two phases and characterize their critical behavior numerically. We extrapolate these quantities to the N→∞ limit (*SI Appendix*).

In Fig. 1, we observe the same two phases as above: The Mott phase is signified by integer $n_{B}$ with $\Psi^{2}=0$ and S/N=0, and the superfluid phase is signified by generic $n_{B}$ and $\Psi^{2}\neq 0$. At the phase boundary, S/N attains a maximum, clearly demarcating the two phases. The phase diagram in Fig. 1 qualitatively matches the equilibrium phase diagram with its $n_{B}=1$ Mott lobe. If M>2, then we would see additional Mott lobes as well (*SI Appendix*).

**Fig. 2.**
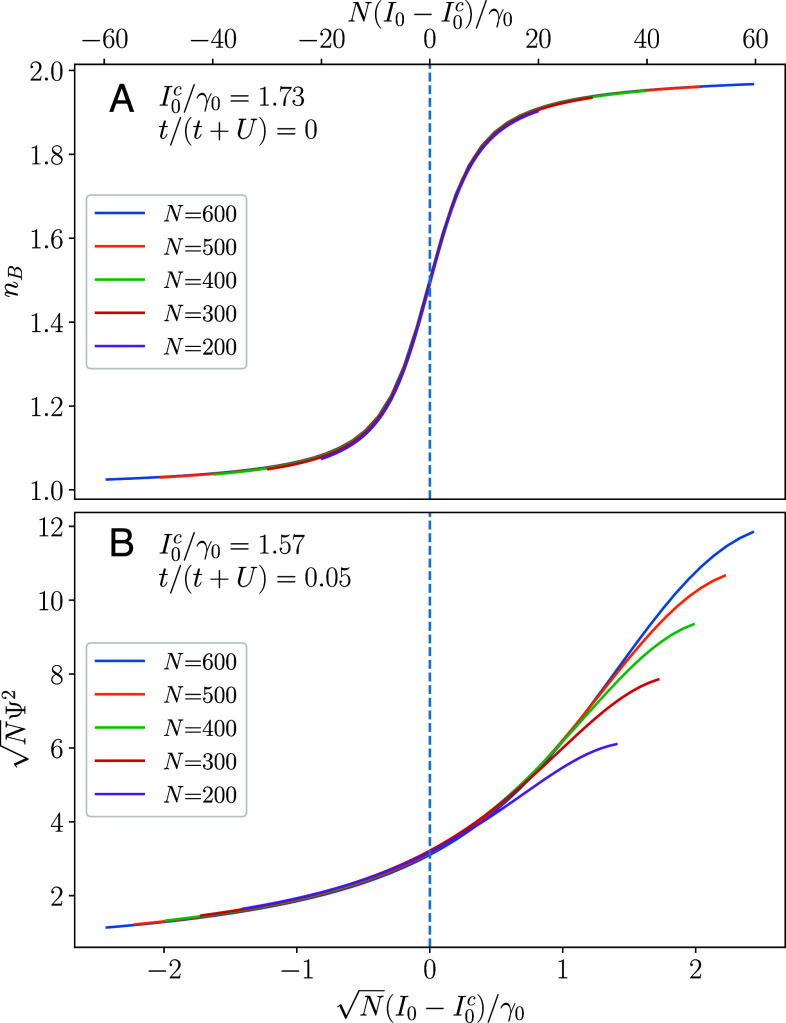
We perform scaling collapses for the order parameters at the (*A*) transition between the $n_{B}=1$ and $n_{B}=2$ Mott phases and (*B*) transition between Mott and superfluid phases. As N→∞, the data in (*A*) [(*B*)] collapses to the functions $f_{n_{B},m}\left(x\right)$ [$f_{\Psi^{2},s}\left(x\right)$] defined in the text. We find that $\lambda_{m}=1$ and $\beta_{m}=0$ for the former and $\lambda_{s}=2=\beta_{s}+1$ for the latter. We have confirmed that this exponent stays the same regardless of which Mott lobes are involved and regardless of whether we tune $I_{0}/\gamma_{0}$ or t/U.

In addition to mapping out the phase diagram, we can confirm the universal scaling forms discussed above. In Fig. 2*A*, at the transition between two Mott phases, we observe that $n_{B}=f_{n_{B},m}\left(N\left(I_{0}-I_{0}^{c}\right)\right)$, as expected from Eq. **13**, indicating that $\lambda_{m}=\beta_{m}+1=1$. Within the equilibrium phase diagram, this transition is first order (a level crossing) indicating that the environment can change the nature of the critical point. In Fig. 2*B*, we similarly see $\Psi^{2}=N^{-1/2}f_{s}\left(N^{1/2}\left(I_{0}-I_{0}^{c}\right)\right)$, as above, for a transition between the Mott and superfluid phase with $\lambda_{s}=2$ and $\beta_{s}=1$, as opposed to the equilibrium values of $\lambda_{s}=\beta_{s}=1$ (*SI Appendix*). We confirm that the same exponents are extracted while tuning t/U instead of $I_{0}/\gamma_{0}$ across the phase transition.

Finally, when M=1, we can extract the Liouvillian gap, $\Delta_{L}$. Away from the critical point, ΔL/N approaches a constant indicating an effect similar to superradiance (54), which is balanced by superabsorption (see below). At criticality, however, we find $\Delta L/N∼N^{-1/\lambda }$, indicating a critical slowing down (i.e. a longer time to reach the steady state) near the phase boundary (*SI Appendix*, Fig. S7). We expect that this same behavior should arise at the phase transitions when M≥2, but we are unable to compute ΔL for large enough system sizes.

## 5. Discussion and Conclusion

We have introduced a solvable variant of the Bose Hubbard model coupled to an environment. As opposed to the zero-temperature equilibrium case where the ground state determines the density matrix, for the nonequilibrium system we focus on the steady-state density matrix. Due to the lack of energy conservation for the open system, the steady state is instead determined by a balance between the radiative decay and (incoherent) laser pumping. We find two phases that resemble the equilibrium Mott and superfluid phases, but the density matrix does not take a Boltzmann form (see e.g. Eq. **27**). We extract critical exponents $\lambda_{m}=\beta_{m}+1=1$ ($\lambda_{s}=\beta_{s}+1=2$) for the transition between a Mott phase and a Mott (superfluid) phase, respectively. These exponents are different from the observed equilibrium exponents derived in *SI Appendix* (and the Mott–Mott transition is first order in equilibrium). The change in criticality is not surprising—although previous work found that the open system criticality can only change a dynamic exponent (32, 46), universality is typically only insensitive to local perturbations, and the environment acts as a global perturbation.

We derive the Lindblad operators and master equations from an explicit form of the coupling between the system and environment. Our model is solvable because the environment couples in a site-invariant way that allows us to only consider the fully symmetric sector of the permutation group, and the calculation is controlled by 1/N for large system size. We note that, although the full eigensystem information of $H_{S}$ is difficult to obtain, only the eigenstates that are connected by the derived Lindblad operators are relevant, and we demonstrate this idea for the case U/t=0 in *SI Appendix*. For generic t/U, it may be possible to obtain Lindblad operators perturbatively controlled by 1/N, but we leave this work for the future.

Our formulation of site-invariant coupling between the system and its environment is reminiscent of the Dicke model (57) and will display superradiance (54). However, since we model the production as the reverse process of decay, our model also has superabsorption. This observation explains why, when I<γ, the steady state has only a finite number of excitons in the thermodynamic limit: if the production rate I is less than the decay rate γ, an extensive number of excitons cannot be built up.

In the experimental systems (27–31), there may be an additional local coupling $H_{I}^{\prime }=\sum_{i}b_{i}^{†}R_{i}^{\prime }+\;\text{h.c.}$ responsible for the production of the bosons, but introducing such a term into our formalism would lead to a sum over site-dependent dissipative terms in the Lindblad equation that requires considering sectors outside of the fully symmetric one. Therefore, the formalism in this paper would be insufficient to solve for the steady state for similarly sized systems. Future work should consider the competition between a local production of bosons and their global (or longer-range) decay, as well as a short-range hopping system Hamiltonian, which might be resolved by numerical methods such as dynamical mean-field theory.

## 6. Materials and Methods

We implement numerically finding the steady state in Python. We first specify a total number of sites N, after which we can loop through all possible $\vec{n}$ and sort them into their corresponding $N_{B}$ sector using $N_{B}=\sum_{m}mn_{m}$. We then specify t, U, and μ and construct the Hamiltonian $H_{0}$ in each $N_{B}$ sector using Eq. **7**. We diagonalize $H_{0}$ using built-in numpy routines and then compute $B_{\alpha ,\beta ,N_{B}}$ using Eq. **8** and the eigenvectors from each sector.

By specifying γ(ω) and I(ω) functions (as in the main text), we then construct a sparse matrix representing the Liouvillian operator, L, acting on the vector space of all possible density matrices. That is, $\dot{\rho }=L\rho$ with L being the right-hand-side of Eq. **10**. Using the sparse matrix routines available in scipy, we can find the eigenvalue–eigenvector pairs with eigenvalues close to zero (or we can directly diagonalize the entire matrix if it is small enough). We have then computed the unique $\rho_{\mathrm{ss}}$ satisfying $L\rho_{\mathrm{ss}}=0$ and the Liouvillian gap $\Delta_{L}$, as defined in the main text. For M>1, we find considerable speed up of the code by only targeting a few eigenvalue–eigenvector pairs which makes the determination of $\Delta_{L}$ difficult. All other quantities we discuss can be computed using $\rho_{\mathrm{ss}}=\sum_{\alpha ,N_{B}}|\alpha N_{B}\rangle \langle \alpha N_{B}|\rho_{ss,\alpha ,N_{B}}$:

[30] $$\begin{array}{c} n_{B}=\frac{1}{N}\left\langle N_{B}\right\rangle =\frac{1}{N}\sum_{\alpha ,N_{B}}N_{B}\rho_{ss,\alpha ,N_{B}} \end{array}$$

and

[31] $$\begin{array}{c} \Psi^{2}=\frac{\left\langle B^{†}B\right\rangle }{N^{2}}=\frac{1}{N^{2}}\sum_{N_{B},\alpha ,\beta }\rho_{ss,\alpha ,N_{B}}{\left|B_{\left(\beta ,\alpha ,N_{B}\right)}\right|}^{2}. \end{array}$$

## Supplementary Material

Appendix 01 (PDF)

## Data Availability

The data, code used to produce the data, and code used to produce the figures will be made available after acceptance at osf.io/cdajz (64).

## Footnotes

^*^We additionally consider U=0 in *SI Appendix* but our formalism must be slightly modified to admit a consistent calculation.

^†^The realistic I(ω) would require sophisticated modeling since the excitons are generated through complicated pump and relaxation processes.
