# Supplementary material for: A solvable model for strongly interacting nonequilibrium excitons
Source: Proc Natl Acad Sci U S A. 2025 Mar 14;122(11):e2424663122. doi: 10.1073/pnas.2424663122 (PMC11929435; doi:10.1073/pnas.2424663122)
Supplement: Supplementary file 1 — Appendix 01 (PDF) [file pnas.2424663122.sapp.pdf]

Supporting Information for

A solvable model for strongly interacting nonequilibrium excitons

Zhenhao Song, Tessa Cookmeyer, and Leon Balents

Leon Balents
E-mail: balents@ucsb.edu
Tessa Cookmeyer
E-mail: tcookmeyer@ucsb.edu

This PDF file includes:

Supporting text
Figs. S1 to S11
SI References

Supporting Information Text

1. Condition for diagonal steady state

Given a generic Hamiltonian $H = H_S + H_R + \sum_\alpha S_\alpha \otimes R_\alpha$, with S_α a system operator and $\mathcal{R}_\alpha$ a reservoir operator, we can separate it into a system (H_S), reservoir (H_R), and interaction piece. The resulting master equation for the density matrix of the system has the form in the weak-coupling limit (1)

$$\dot{\rho}_S = -i[H_S + H_{LS}, \rho_S] + \sum_{\omega,\alpha,\beta} \gamma_{\alpha,\beta}(\omega)\mathcal{L}\left(S_\beta(\omega), S^\dagger_\alpha(\omega)\right)[\rho] \quad [1]$$

where $S_\alpha(\omega) = \sum_{E'-E=\omega} \Pi(E)S_\alpha\Pi(E')$ with $\Pi(E)$ the projector on the energy E eigenspace of H_S, and $H_{LS} = \sum_E \Pi(E)H_{LS,E}\Pi(E)$ is the Lamb shift Hamiltonian. As before $\gamma_{\alpha,\beta}(\omega)$ is expressed as correlators of the $R_{\alpha,\beta}$.

We now will assume that the density matrix only has coherences within each eigensector and show that it remains in this form under time evolution. To do so, we write the density matrix as $\rho_S = \sum_E \Pi(E)\rho(E)\Pi(E)$ where $\rho(E)$ is density matrix acting on the energy E eigenspace. For this form, we can explicitly compute

$$\dot{\rho}_S = -i[H_S + H_{LS}, \rho_S] + \mathcal{D}[\rho] = \sum_E \Pi(E)[H_{LS,E}, \rho(E)]\Pi(E) + \mathcal{D}[\rho] \quad [2]$$

with dissipative part

$$\begin{aligned}\mathcal{D}[\rho] = \sum_{\omega,\alpha,\beta} \gamma_{\alpha,\beta}(\omega) \sum_{E'-E=\omega} \Big(&\Pi(E)S_\beta\Pi(E')\rho(E')\Pi(E')S^\dagger_\alpha\Pi(E) \\ &-\frac{1}{2}\Pi(E')S^\dagger_\alpha\Pi(E)S_\beta\Pi(E')\rho(E')\Pi(E') - \frac{1}{2}\Pi(E')\rho(E')\Pi(E')S^\dagger_\alpha\Pi(E)S_\beta\Pi(E')\Big)\end{aligned} \quad [3]$$

We therefore see that the density matrix remains block diagonal in the different energy sectors. We can always search for a steady state of this form, especially since we can always start from a ground state. We then turn on incoherent pumping, which will never create coherences between different energy blocks. The only way to create and maintain such coherences in this limit would be through a time-dependent H_S.

An immediate consequence of this result is that if the spectrum of H_S is non-degenerate, then the steady-state density matrix will be diagonal, $\rho = \sum_E \rho_E |E\rangle\langle E|$. However, this condition is not necessary.

In our above analysis, we always have diagonal damping rates $\gamma_{\alpha,\beta}(\omega) = \gamma_{\alpha,\alpha}(\omega) = \gamma_\alpha(\omega)$ with $\gamma_+(\omega) = I(\omega)$ $[\gamma_-(\omega) = \gamma(\omega)]$ for corresponding operator $S_+ = \mathcal{B}^\dagger$ ($S_- = \mathcal{B}$), respectively. To find a diagonal ρ in this case, we just need that in some basis of each eigensector, $S_\pm(\omega)|E, m\rangle \propto |E \pm \omega, k\rangle$ (where m, k are indexing the degeneracy), i.e. the choice of basis leads to jump operators that do not create coherences between states of the same energy. This condition ensures that a density matrix $\rho = \sum_{E,m} \rho_{E,m}|E, m\rangle\langle E, m|$ stays of this form. Notably, since there is a conserved quantity N_B in our model that is changed by the $S_\pm$, it is clear that this condition will always hold if there is no degeneracy within each fixed N_B sector. If there is such degeneracy, the density matrix will still have a diagonal form if $S_\pm(\omega)$ does not generate superpositions of degenerate states. Writing this condition in the notation of the main text gives the result as stated there.

2. Standard assumption of Lindblad operators

Let's analyze the case with $t = 0$ with the form of the jump operators from e.g. Refs (2, 3). We have

$$\begin{aligned}H_0 &= \mu \sum_i \Big(n_i + \frac{U}{2}n_i(n_i - 1)\Big) \\ \dot{\rho}(t) &= -i[H_0, \rho] + \sum_i \Big(\gamma\mathcal{L}(b_i, b^\dagger_i)[\rho] + I\mathcal{L}(b^\dagger_i, b_i)[\rho]\Big)\end{aligned} \quad [4]$$

In this case, the sites are decoupled and $\rho = \bigotimes_i \rho_i$. We can therefore consider only one site at a time. We write out the density matrix in the number basis $\rho_i = \sum_{n=0}^M \rho_{i,n}|n_i\rangle\langle n_i|$, whose form is preserved under time evolution, and we find

$$\dot{\rho}_{i,n} = (n + 1)(\gamma\rho_{i,n+1} - I\rho_{i,n}) + n(I\rho_{i,n-1} - \gamma\rho_{i,n}). \quad [5]$$

As in the main text, we see that the form $\rho_{i,n} = \rho_{i,0}(I/\gamma)^n$ is a solution.

There are at least two shortcomings to this formalism, however, that are apparent even in this simple example: first, this steady state appears to not depend at all on the parameters of H_0 (as opposed to our solution where U/μ affects the location of the transitions). Second, it is not clear how to include temperature. If we assume that the bath has a temperature T, there is no clear way to include it in the definition of I and γ because they lack a dependence on the energy. One natural way would be to assume $I/\gamma = e^{-\mu/T}$, but the fact that the energy is quadratic in n means that the steady state cannot fit the thermal form $\rho_n = e^{-(\mu n+Un(n-1)/2)/T}$. As we discuss in the main text, our approach readily capture the coupling to a thermal reservoir, which leads to the steady-state density matrix being of the Boltzmann form $\rho \sim e^{-H/T}$, a well-known attribute of the weak-coupling limit Lindblad formalism (1).

3. Short-ranged or long-ranged Lindblad operators?

In our text, we assumed that the wavelength of light was much larger than the extent of the system and therefore we took a translation-invariant coupling to our sites. In most other work, e.g. (2, 3), the opposite limit is chosen. How do we know which limit is applicable and/or how do we extrapolate between them?

The derivation of the master equation provides the answer. Let's assume the environment is solely composed of photons and the system and environment couple in the natural way, as before (4)

$$H_I = \sum_{\boldsymbol{k},\pm} g_{\boldsymbol{k},\pm} a_{\boldsymbol{k},\pm} b_{\boldsymbol{k}}^\dagger + \text{H.c.} = \sum_i \mathcal{R}_i b_i^\dagger + \text{H.c.} \qquad [6]$$

where $\mathcal{R}_i = \frac{1}{\sqrt{N}} \sum_{\boldsymbol{k},\pm} g_{\boldsymbol{k},\pm} a_{\boldsymbol{k},\pm} e^{i\boldsymbol{x}_i \cdot \boldsymbol{k}}$.

Regardless of what the system Hamiltonian is (assuming it satisfies the assumptions needed for the derivation of the master equation), the environment will only enter through the computation of

$$\gamma_{i,j}(\omega) = \int_{-\infty}^{\infty} ds e^{i\omega s} \langle \mathcal{R}_i(s) \mathcal{R}_j^\dagger \rangle; \qquad I_{i,j}(\omega) = \int_{-\infty}^{\infty} ds e^{i\omega s} \langle \mathcal{R}_i^\dagger(s) \mathcal{R}_j \rangle \qquad [7]$$

The computation is almost identical, so we just focus on

$$\gamma_{i,j}(\omega) = \frac{1}{N} \sum_{\boldsymbol{k},\pm} 2\pi\delta(\omega - k) |g_{\boldsymbol{k},\pm}|^2 e^{i(\boldsymbol{x}_i - \boldsymbol{x}_j)\cdot \boldsymbol{k}} n_k \qquad [8]$$

where we assumed that $H_R = \sum_{\boldsymbol{k},\pm} k a_{\boldsymbol{k},\pm}^\dagger a_{\boldsymbol{k},\pm}$ and a thermal distribution of light with $\langle a_{\boldsymbol{k},\pm}^\dagger a_{\boldsymbol{k},\pm} \rangle = n_k$. In order to evaluate further, we need to know what the $g_{\boldsymbol{k},\pm}$ are, which depend on the details of the system. Here we just assume it varies smoothly with $\boldsymbol{k}$. We note that, when $|\boldsymbol{x}_i - \boldsymbol{x}_j| k \gg 1$, with k set by ω, the angular integral will lead to terms that average to zero for $i \neq j$, and thus the dissipative part takes the form $\sum_i \gamma \mathcal{L}(L_i, L_i^\dagger)$, i.e. local pump and decay apply here. However, when $|\boldsymbol{x}_i - \boldsymbol{x}_j| k \ll 1$, it is clear that the jump operators arising from $\mathcal{R}_i$ and $\mathcal{R}_j$ will mix together, and then we have a dissipative term of the form $\gamma \mathcal{L}(\sum_i L_i, \sum_j L_j^\dagger)$. In the latter case, we just recover the situation considered in the main text, i.e. a global pump and decay.

4. Analytic solution of $U/t = 0$ limit

As we stated in the text, the full eigensystem information of H_S can be hard to obtain, but only the eigenstates that are connected by the derived Lindblad operators are relevant. Here we demonstrate this idea in the $U/t = 0$ limit of the Bose Hubbard model, where we can also obtain an anlytical solution of the steady state. In this case, it's better to reformulate the system Hamiltonian as a spin-$M/2$ system, so that we can have a direct comparison with the numerics which imposes a boson number truncation M. To do so, we just need to redefine the action of b_i in H_0 by

$$b_i|n_i\rangle = \sqrt{n_i(M+1-n_i)}|(n-1)_i\rangle; \qquad b_i^\dagger|n_i\rangle = \sqrt{(n+1)(M-n)}|(n+1)_i\rangle; \qquad \hat{n}_i|n_i\rangle = n_i|n_i\rangle. \qquad [9]$$

These map to spin-$M/2$ operators $b_i^\dagger \to S_i^+$, $b_i \to S_i^-$, and $\hat{n}_i \to S_i^z + M/2$ satisfying the usual commutation relations. We then have the system Hamiltonian

$$H_s = \mu \sum_i \hat{n}_i - \frac{t}{N} \sum_{i,j} b_i^\dagger b_j \qquad [10]$$

We still define the coupling operator $\mathcal{B} = \sum_i b_i$. One can then verify the commutation relation:

$$[H_s, \mathcal{B}] = (-\mu + Mt - \frac{2t}{N} \hat{N}_B)\mathcal{B}. \qquad [11]$$

Note that $\mathcal{B}$ is not an eigenoperator since $\hat{N}_B$ is an operator. But we can decompose $\mathcal{B}$ as

$$\mathcal{B} = \sum_{N_B} \mathcal{B}_{N_B} \qquad [12]$$

where $\mathcal{B}_{N_B} \equiv P_{N_B - 1} \mathcal{B} P_{N_B}$, and P_{N_B} is the projector onto the sector of boson number N_B. Then, one can verify

$$[H_s, \mathcal{B}_{N_B}] = (-\mu + Mt - 2t\frac{N_B - 1}{N})\mathcal{B}_{N_B} \qquad [13]$$

and thus $\mathcal{B}_{N_B}$ are eigenoperators, with energies $\omega_{N_B} = \mu - t(M - \frac{2(N_B-1)}{N})$, which have the same form as in the $M = 1 (U/t \neq 0)$ case in the text. In the $M = 1 (U/t \neq 0)$ case, each N_B sector has only one eigenstate and thus we can label them by $|N_B\rangle$ and write down the master equation in such basis. Here in each N_B sector, there are clearly more than one eigenstate. However, since we start from the vacuum state, all the states visited during the Lindblad time evolution are those generated by the Lindblad operators, i.e. we may still denote $|N_B\rangle \equiv C_{N_B} (\mathcal{B})^{N_B} |\text{vac}\rangle$, with C_{N_B} being the normalization factor. One can check that $\mathcal{B}(\mathcal{B}^\dagger)$ acting on the normalized $|N_B\rangle$ gives us $\sqrt{N_B(NM - 2N_B)}|N_B - 1\rangle \left(\sqrt{(N_B+1)(NM - 2N_B)}|N_B + 1\rangle\right)$. Then, we can write down the master equation accordingly, and the solution would have the same structure as in $M = 1(U/t \neq 0)$.

We compare the analytical solution with the numerics in Fig. S1. The alignment between them confirms our arguments above.

5. Derivation of $M = 1$ density matrices and observables

We first consider the Mott phase, where $r_< = I(\mu - t)/\gamma(\mu - t) \ll 1$ or $r_> = I(\mu + t)/\gamma(\mu + t) \gg 1$. For $r_< < 1$, from Eq. (26) in the main text, we have

$$\rho_m = \rho_0 \prod_{j=1}^{m} r(\omega_j) = \rho_0 r_<^m \prod_{j=1}^{m} \left(1 + \frac{r'_<}{r_<}\frac{2tj}{N}\right) \approx \rho_0 r_<^m \exp\left(\sum_{j=1}^{m} \frac{2r'_< t}{r_< N} j\right) \approx \rho_0 r_<^m \exp\left(\frac{r'_< t}{r_< N} m^2\right)$$

where we Taylor expand r_j, convert the product to the exponential of a sum, and keep only the leading order term in a $1/N$ expansion of the exponential. The weight ρ_m/ρ_0 at large m is suppressed by $r_<^m$ as well as the exponential function. This calculation is controlled because $m \lesssim \sqrt{N}$ are the only values where ρ_m is substantial (if $r_< \leq 1$).

When $r_< \ll 1$, ρ_m decays very fast and we are in the deep Mott phase. Note that $r'_< < 0$ is exactly our assumption that $r(\omega)$ monotonically decreases, as stated in the text. Similarly, we can consider the case $r_> \gg 1$, and we get

$$\rho_{N-m} = \rho_N \prod_{j=0}^{m-1} [r(\omega_{N-j})]^{-1} \approx \rho_N r_>^{-m} \exp\left(\frac{r'_> t}{N} m^2\right) \qquad [14]$$

Next, we analyze the intermediate phase, when $r_< > 1 > r_>$ and $r_{j^*} = 1$ with $j^*/N \sim O(1)$. An almost identical calculation gives

$$\rho_{j^*+m} = \begin{cases} \rho_{j^*} \prod_{j=0}^{m} \left(1 + r'_{j^*} \frac{2tj}{N}\right) & m \geq 0 \\ \rho_{j^*} \prod_{j=0}^{-m} \left(1 - r'_{j^*} \frac{2tj}{N}\right)^{-1} & m \leq 0 \end{cases} \approx \rho_{j^*} \exp\left(\frac{r'_{j^*} t}{N} m^2\right) \qquad [15]$$

where we used $r_{j^*} = 1$, and thus we have only an exponential decay term compared to the Mott phase, which would be slower since the exponent is suppressed by $1/N$. This holds true until m^2 reaches N, where the density matrix weight is already negligible. Therefore, we should have $m \lesssim \sqrt{N}$. For j^* near 0 or N, $j^* \pm m$ should be truncated by 0 and N, respectively.

Now, we can calculate the observables and critical behaviors. Deep inside the superfluid phase, we have the order parameter $\Psi^2 = \langle \mathcal{B}^\dagger \mathcal{B} \rangle / N^2$

$$\Psi^2 = \frac{\sum_m (j^* + m)(N - j^* - m + 1) \exp\left(\frac{r'_{j^*} t}{N} m^2\right)}{N^2 \left(\sum_m \exp\left(\frac{r'_{j^*} t}{N} m^2\right)\right)} \approx \frac{j^*}{N}\left(1 - \frac{j^*}{N}\right) + O(1/\sqrt{N}) \qquad [16]$$

where m is summed from $-j^*$ to $N - j^*$ but only the terms with $|m| \lesssim \sqrt{N}$ contribute. We keep only the leading order terms.

From the Mott phase side, we can calculate Ψ^2 as well. We assume $r_< \ll 1$, we can ignore the exponential corrections and evaluate

$$\Psi^2 = \frac{\sum_{m=0}^{N} m(N - m + 1) r_<^m \exp\left(\frac{r'_< t}{r_< N} m^2\right)}{N^2 \left(\sum_{m=0}^{N} r_<^m \exp\left(\frac{r'_< t}{r_< N} m^2\right)\right)} \approx \frac{1}{N}\left(\frac{\sum_m m r_<^m}{\sum_m r_<^m}\right) = \frac{1}{N}\frac{r_<}{1 - r_<} \qquad [17]$$

A similar calculation for $r_> \gg 1$ holds and we find $\Psi^2 \approx (1 - r_>^{-1})^{-1}/N$.

Finally, if we approach the phase transition from the left, $r_< \to 1^-$, we use $r_<^m = e^{\ln(r_<^m)} \approx e^{m(r_< - 1)}$ to write

$$\Psi^2 \approx \frac{1}{N^2} \frac{\rho_0 \sum_{m=0}^{N} m(N - m + 1) e^{\frac{r'_< t}{r_<}\frac{m^2}{N} + m(r_< - 1)}}{\rho_0 \sum_{m=0}^{N} e^{\frac{r'_< t}{r_<}\frac{m^2}{N} + m(r_< - 1)}} \approx \frac{1}{N^{1/2}} \frac{\int_0^{\sqrt{N}} dx x \exp\left[\frac{r'_< t}{r_<} x^2 - (1 - r_<)\sqrt{N} x\right]}{\int_0^{\sqrt{N}} dx \exp\left[\frac{r'_< t}{r_<} x^2 - (1 - r_<)\sqrt{N} x\right]}.$$

where we changed to the variable $x = m/\sqrt{N}$ and approximated the sums by integrals. The integrand quickly decays when $x \gtrsim 1$ so we can extend the limits of integration to infinity, and we then get the scaling form $\Psi^2 = N^{-1/2} f_s(N^{1/2}(1 - r_<))$. We verified that the same scaling form applies when we approach the phase transition from either side, and $n_B = \langle N_B \rangle / N$ has a scaling form with the same critical exponents as well.

6. Extrapolation details

In our numerical calculations, the lattice size can be up to 1000 for $M = 2$ and 100 for $M = 3$, within our computational capability. To get the phase diagram shown in the text, we extrapolate the data at each point of t/U and I_0/γ_0 of several system sizes to thermaldynamic limit ($N = \infty$). We use a polynomial extrapolation of order 2 (quadratic fitting), which is equivalent to assuming $O(N) = O(N = \infty) + O' \frac{1}{N} + O'' \frac{1}{N^2} + ...$ and truncate it up to the second order of $1/N$. We find that the truncation up to quadratic order already gives us fair results. The fitting detail is shown in Fig. S2, where not all finite N curves are plotted. For the extrapolation of the entropy, we get negative extrapolated entropy values at negligibly few points. We manually set them to be 0 since a negative entropy is not physical and we believe this abnormal behavior is a numerical artifact during the extrapolation. We also tried a power law fitting in $1/N$, which gives almost the same results but fails near the phase transitions. We adopt the polynomial fitting results for it gives a more smooth behavior globally.

7. Phase diagrams

In the text we showed the phase diagram in terms of Ψ^2. Here we show the phase diagrams mapped in terms of n_B and S/N in Fig. S3. We see that the extracted phase boundaries from the entropy clearly delineate the Mott regions seen in the n_B plot.

We also show the phase diagram for $M = 3$, by plotting Ψ^2 and n_B vs. I_0/γ_0 and $t/(t+U)$ in Fig. S4. In this case, we can only reach up to $N = 70$, but we can already see the Mott insulating domes appearing. The shape of the $n_B = 2$ lobe is clearly very different from the $M = 2$ case, but the $n_B = 1$ lobe appears very similar. This plot demonstrates that the effect of the truncation is unlikely to affect the physics near the $n_B = 1$ lobe.

8. Critical scaling

A. Mott-Mott transition. In the main text, we studied n_B, the order parameter for characterizing the Mott-Mott transition point, and confirmed the critical scaling form $n_B = f_{n_B,m}(N(I_0 - I_c))$. In Fig. S5 we show the scaling collapse for Ψ^2 at this transition point, and the same scaling form as n_B also applies here i.e. $\Psi^2 = f_{\Psi^2,m}(N(I_0 - I_c))$.

B. Mott-Superfluid transition. In the main text, we displayed the critical scaling from Mott to superfluid phases by tuning $r = I_0/\gamma_0$, where we confirmed the scaling form $\Psi^2(N) = N^{-1/2} f_{\Psi^2,s}(\sqrt{N}(r - r_c))$. In Fig. S6 we show that this scaling form applies if we tune t/U at fixed I_0/γ_0 instead, and, in both cases, the same exponents are found.

We expect that this scaling form should almost everywhere at the Mott-superfluid phase boundary. In the equilibrium case, there are two different universality classes for the Mott-superfluid transition depending on whether the density remains at $n_B \in \mathbb{Z}$ (at the peak of the Mott lobe) or if the density is generic (5). The two can be distinguished as having different values of the dynamic critical exponent, z. In principle, the Liouvillian gap, described in the next section, determines how quickly the system approaches the steady state and could probe z. In practice, we can only reach the scaling regime for the Liouvillian gap for $M = 1$, which does not have the “special” critical point as there is no peak of the Mott lobe.

C. Liouvillian gap. The Liouvillian, $\mathcal{L}$, is the superoperator defining the right-hand-side of the master equation $\dot{\rho} = \mathcal{L}\rho$. Here we measure the Liouvillian gap, $\Delta_{\mathcal{L}}$ defined as the smallest real part of the non-zero eigenvalues of $\mathcal{L}$ (6). This gap determines the characteristic time for the system to reach its steady state. In Fig. S7, it's clear that $\Delta_{\mathcal{L}}$ reaches a minimum at the (Mott-superfluid) phase boundary, which implies a critical slowing down near the phase transitions. As in the superradiance phenomenon, the system decay rate scales with N(1), so we rescaled $\Delta_{\mathcal{L}}/N$ to make it clear that there is a slowing down at the phase transition relative to the rest of the phase diagram.

In Fig. S8, we perform a scaling collapse for $\Delta_{\mathcal{L}}$ with $M = 1$. In equilibrium, the energy gap $\Delta E \sim (t - t_c)^\delta$ where $\delta = \nu z$ for z the dynamic critical exponent and ν the correlation length exponent. In the mean-field theory of the equilibrium Bose-Hubbard model $\delta = 1$. Similarly, we see that $\Delta_{\mathcal{L}}/N = N^{-\delta'/\lambda} g_{\mathcal{L}}(N^{1/\lambda}(I_0 - I_c))$ for some scaling function $g_{\mathcal{L}}$ and exponent δ', λ (that are potentially different between the Mott-Mott and Mott-superfluid transition) implying that $\Delta_{\mathcal{L}}/N \sim (I_0 - I_c)^{\delta'}$ close to the transition. We numerically observe $\delta'_s = \delta'_m = 1$ (as well as $\lambda_m = \lambda_s - 1 = 1$ as before) similar to $\delta_s = 1$ for the equilibrium transition. We consider $\Delta_{\mathcal{L}}/N$ instead of $\Delta_{\mathcal{L}}$ so that a finite value is reached away from the critical point in the thermodynamic limit.

Calculations of $\Delta_{\mathcal{L}}$ for $M \geq 2$ are beyond our computational capabilities, but we expect that the slowing-down and scaling form do not depend on the truncation M and will hold for general M.

9. Steady state distributions

It's worth noting that in our model, the resulting steady states are non-thermal. In Fig. S9, we display the steady state density matrix distribution with respect to system energy obtained by numerics at a generic point in the phase space ($I_0/\gamma_0 = 1.26, t/(t+U) = 0.1$), and $M = 2$. In the inset, we can see a oscillation feature within each N_B sector, which emphasizes the non-thermal property of the steady state. The peak structure of $\rho(E)$ cannot be explained through a grand canonical form for $\rho(E) \sim e^{-\beta(E-\mu N)}$.

10. Comparison with equilibrium system

In Fig. S10, we show the (zero-temperature) equilirbium phase diagram of the all-to-all hopping Bose Hubbard Hamiltonian, in terms of $t/(t+U)$ and $\mu/(t+U)$, where μ is the bosonic chemical potential. The nonequilibrium and equilibrium phase diagrams have similar shapes, which agrees with our intuition that increasing the intensity of the light is analogous to changing the chemical potential.

However, there are key differences: First, at $t/(t+U) = 0$, the order parameter Ψ^2 remains zero at any μ. This is different than the non-equilibrium case, where Ψ^2 has a peak and shows critical scaling at the transition point of two Mott lobes. Second, at any point in the phase diagram, the observables ($\Psi^2 = \langle \mathcal{B}^\dagger \mathcal{B} \rangle / N^2$ and $n_B = \langle N_B \rangle / N$) do not scale with N when N is large enough, which is reasonable since the all-to-all hopping is equivalent to enforcing a mean field behavior. Third, the entropy remains zero in the whole phase diagram, because at zero temperature, we are always at the ground state, which in general has no degeneracy. However, we observe a peak of entropy at the non-equilibrium phase transition.

Finally, we comment on the critical exponents. For the equilibrium Mott-Mott transition, we can only tune μ to move directly from e.g. the $n_B = 0$ to the $n_B = 1$ lobe. The transition is simply between the eigenstate with every site having 0

bosons and the eigenstate with every site having 1 bosons. By analyzing the energy per site, it is clear that this transition is first-order. In the nonequilibrium case, we find that this transition has become second order with an order parameter, e.g. $n_B = \Theta(I_0 - I_c)$ for the $n_B = 0$ to $n_B = 1$ lobe, that has the finite-size scaling form $n_B = N^{-\beta_m/\lambda_m} f_{n_B,m}(N^{1/\lambda_m}(I_0 - I_c))$ with $\lambda_m = \beta_m + 1 = 1$.

For the Mott-superfluid transition, as we mentioned above, there is a "generic" transition, occurring in most of the phase diagram, and a "special" transition occurring at the peak of the Mott lobe. Both critical points have an energy gap that scales as $\Delta E \sim (t - t_c)^{\nu z}$ with $\nu z = 1$ but the former has $z = 2$ and the latter $z = 1$. We are unsure if we can capture the "special" transition in our nonequilibrium model, but we can determine the critical exponents β_s and λ_s as before for the generic case.

Our all-to-all model mimics the mean-field limit of the Bose-Hubbard model (7), and we can then immediately know that the mean-field value of $\beta_s = 1$ (since our order parameter is Ψ^2 and not Ψ). In fact, we can predict the scaling behaviors through a path integral formulation. The partition function, with an applied external field $(-h^* b_i - h b_i^\dagger)$, is given by

$$Z(h) = \int \mathcal{D}b_i \mathcal{D}b_i^* e^{-\int d\tau \left(-\frac{t}{N} \left(\sum_i b_i^*\right)\left(\sum_j b_j\right) + \sum_i \left(b_i^* \partial_\tau b_i + U n_i^2 - \mu n_i\right) - \sum_i \left(h^* b_i + h b_i^*\right) \right)} \quad [18]$$

$$= \int \mathcal{D}b_i \mathcal{D}b_i^* \mathcal{D}\psi \mathcal{D}\psi^* e^{-\int d\tau \left(\sum_i \left((\psi^* - h^*) b_i + (\psi - h) b_i^* + b_i^* \partial_\tau b_i + U n_i^2 - \mu n_i \right) + \frac{N}{t} |\psi|^2 \right)} \quad [19]$$

In the second line, we make a Hubbard-Stratonovich transformation to decouple the hopping term, and only a single auxiliary field is needed due to the all-to-all hopping form. Since all sites are decoupled, we can integrate out each b_i and arrive at an effective action for field ψ, where each b_i integration gives the same contribution.

$$S_{\text{eff}} = \int d\tau \Big(N f(\psi - h) - \frac{N}{t} |\psi^2| \Big) \quad [20]$$

$$= \int d\tau N \Big(r_0 |\psi - h|^2 + u_0 |\psi - h|^4 - \frac{1}{t} |\psi^2| + ... \Big) \quad [21]$$

$$= \int d\tau N \Big(r_0 (|\psi|^2 - h^* \psi - h \psi^*) + u_0 (|\psi|^4 - 2h^* \psi |\psi|^2 + \text{c.c.}) - \frac{1}{t} |\psi^2| + ... \Big) \quad [22]$$

$$= \int d\tau N \Big(r |\psi|^2 + u_0 |\psi|^4 - r_0 h \psi^* - 2u_0 h \psi^* |\psi|^2 + \text{c.c.} + ... \Big) \quad [23]$$

where $f(x)$ is some function obtained by integrating out b_i field. In the second line we Taylor expand $f(x)$ according to symmetry constraints, and we ignored the Berry phase term $\psi^* \partial_\tau \psi$ as we assume that time-independent fields are most important at the saddle point (7). In the third line we expand $|\psi - h|$ and omit terms of higher orders in h or eventually $1/N$. We define $r \equiv r_0 - 1/t$, and at the critical t_c, r should go to zero, and $r \sim \delta t = t - t_c$ to the lowest order. Now, we rescale $\psi \to \psi/\sqrt{Nr}$. If there is any change in the measure of $\int \mathcal{D}\psi$, it only contributes a constant term $C(N)$ to the free energy. Then, we have

$$S_{\text{eff}} = \int d\tau \Big(|\psi|^2 + \frac{u_0}{Nr^2} |\psi|^4 - \frac{r_0 \sqrt{N}}{\sqrt{r}} h \psi^* - \frac{2u_0}{\sqrt{Nr^3}} h \psi^* |\psi|^2 + \text{c.c.} + ... \Big) \quad [24]$$

$$\sim \int d\tau \Big(|\psi|^2 + \frac{u_0}{N\delta t^2} |\psi|^4 - \frac{r_0 \sqrt{N}}{\sqrt{\delta t}} h \psi^* - \frac{2u_0}{\sqrt{N\delta t^3}} h \psi^* |\psi|^2 + \text{c.c.} + ... \Big) \quad [25]$$

So, the free energy $f = -\frac{1}{\beta N} \ln Z = -\frac{1}{\beta N} \int \mathcal{D}\psi \mathcal{D}\psi^* e^{-S_{\text{eff}}}$ should take the form of

$$N f(N, h) = F\Big(N\delta t^2, h\sqrt{\frac{N}{\delta t}}, \frac{h}{\sqrt{N\delta t^3}}\Big) \quad [26]$$

And we can calculate the order parameter Ψ^2 at zero external field as

$$\Psi^2 = \langle \big(\sum_i b_i^\dagger\big)\big(\sum_j b_j\big) \rangle / N^2 \quad [27]$$

$$= \frac{1}{N^2} \frac{\partial^2}{\partial h \partial h^*} F\Big(N\delta t^2, h\sqrt{\frac{N}{\delta t}}, \frac{h}{\sqrt{N\delta t^3}}\Big)\Big|_{h=0} \quad [28]$$

$$= \frac{1}{N\delta t} G_1\big(N\delta t^2\big) + \frac{1}{(N\delta t)^2} G_2\big(N\delta t^2\big) + \frac{1}{(N\delta t)^3} G_3\big(N\delta t^2\big) \quad [29]$$

where $G_1(x)$, $G_2(x)$ and $G_3(x)$ are different unknown functions. When we perform finite-size scaling collapses, we fix the lattice size N, and examine $\Psi^2(N)$ near the critical point, i.e. $\delta t \sim 0$. Therefore, we should look at Ψ^2 at the limit $\delta t \to 0$.

Because $\Psi^2 \to 0$ at the transition, we must have $G_1(0) = G_2(0) = G_3(0) = 0$. If we assume that $G_i(x)$ can be Taylor expanded at $N\delta t^2 = 0$, we find $G_3'(0) = 0$ and $\Psi^2 = G_1'(0)\delta t + \frac{1}{N}G_2'(0) + ...$ Equivalently, $N\Psi^2 \sim G_1'(0)N\delta t + G_2'(0) + ...$, which is consistent with the scaling collapse we find numerically in Fig. S11 for $M = 1$ and $M = 2$. We thus confirm that $\lambda_s = \beta_s = 1$ at the "generic" transition point in the equilibrium case, in contrast to the nonequilibrium $\lambda_s = \beta_s + 1 = 2$.

As a double check, we can calculate the scaling forms in the case of $M = 1$ where an exact solution can be obtained. Recall that there is only one state in the (fully symmetrized) sector of N_B bosons, labeled as $|N_B\rangle$. We have

$$E_{N_B} = \mu N_B - \frac{t}{N}N_B(N - N_B + 1); \qquad \frac{\mathcal{B}^\dagger\mathcal{B}}{N^2}|N_B\rangle = \frac{N_B}{N}(1 - \frac{N_B}{N} + \frac{1}{N}). \tag{30}$$

By treating $n_B = N_B/N$ as a continuous variable, it is easy to find that the Mott-superfluid transition occurs at $t_c = -\mu/(1-\frac{1}{N}) \sim -\mu$, where we choose $\mu < 0$ to study the $n_B = 1$ to superfluid phase transition. For the $\mu > 0$ transition, the same analysis can be carried out. The order parameter can be obtained as

$$\Psi^2 = \begin{cases} \frac{1}{N} & t < t_c \\ \frac{1}{4} - \frac{\mu^2}{4t^2} + \frac{1}{2N} + \frac{1}{4N^2} & t > t_c \end{cases} \tag{31}$$

And we can expand $t = \delta t - \mu$ near the critical point. After keeping only the lowest order terms of δt (suitable for doing finite size scaling), we get

$$\Psi^2 \sim -\frac{\delta t}{2\mu} + \frac{1}{2N} + \frac{1}{4N^2} \tag{32}$$

This result confirms again that $\lambda_s = \beta_s = 1$, consistent with the result of path integral calculation and our numerical results (Fig. S11).

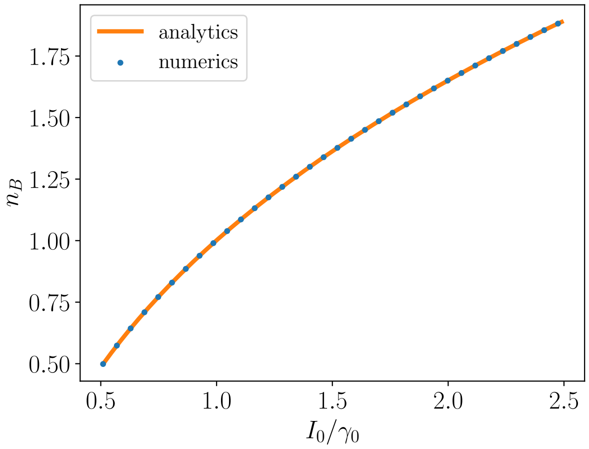

Fig. S1. The analytical and numerical results for $U = 0$.

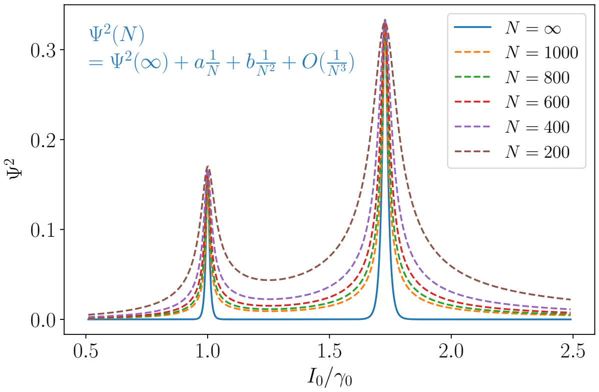

Fig. S2. The details of extrapolation to $N = \infty$ for Ψ^2 for $M = 2$ at $t = 0, U = 30, \mu = 150$. The same procedures are carried out for other t/U values and other observables.

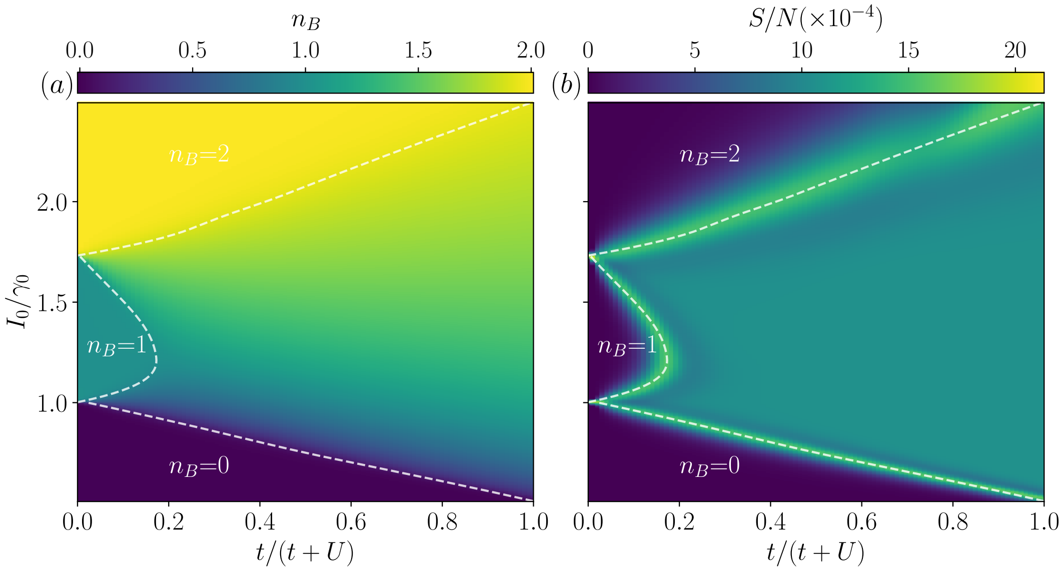

Fig. S3. The phase diagrams mapped in terms of (a) n_B and (b) S/N, for $M = 2$. Every data point is extrapolated to $N = \infty$ as indicated in the Supporting text.

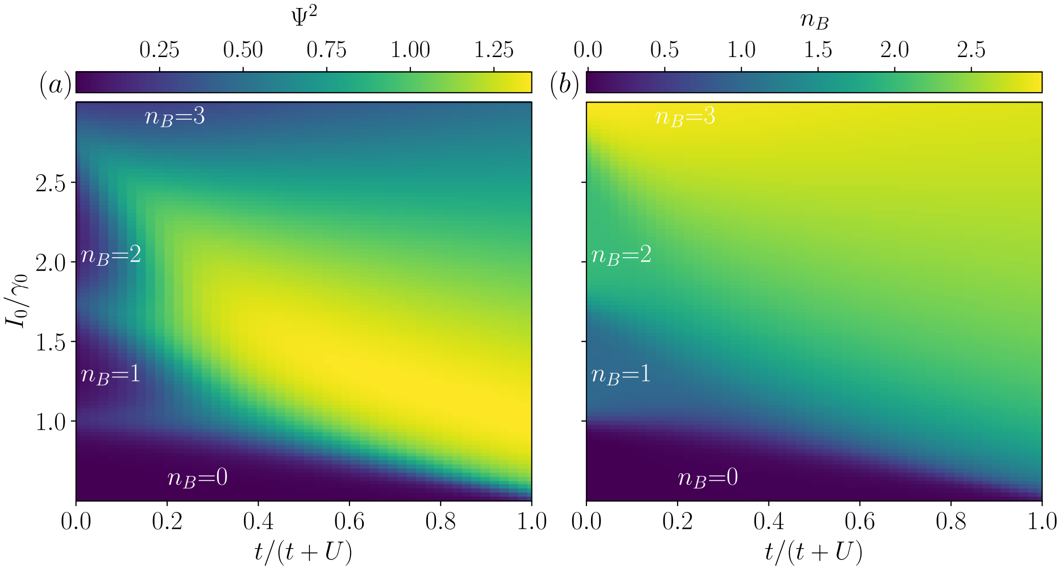

Fig. S4. The phase diagrams mapped in terms of (a) Ψ^2 and (b) n_B, for $M = 3$. We see the Mott lobes appearing; although the $n_B = 2$ region has now become a lobe, the $n_B = 1$ lobe seems almost identical.

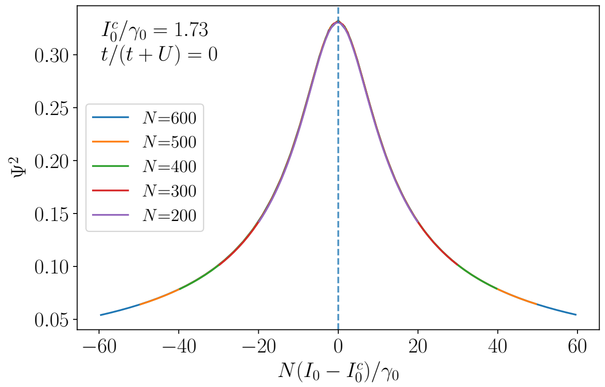

Fig. S5. Scaling collapses for Ψ^2 at the Mott-Mott transition.

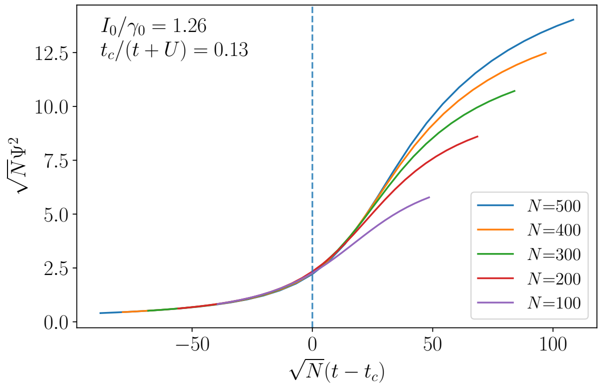

Fig. S6. Scaling collapses at the Mott-superfluid transition, where t/U is tuned and I_0/γ_0 is fixed.

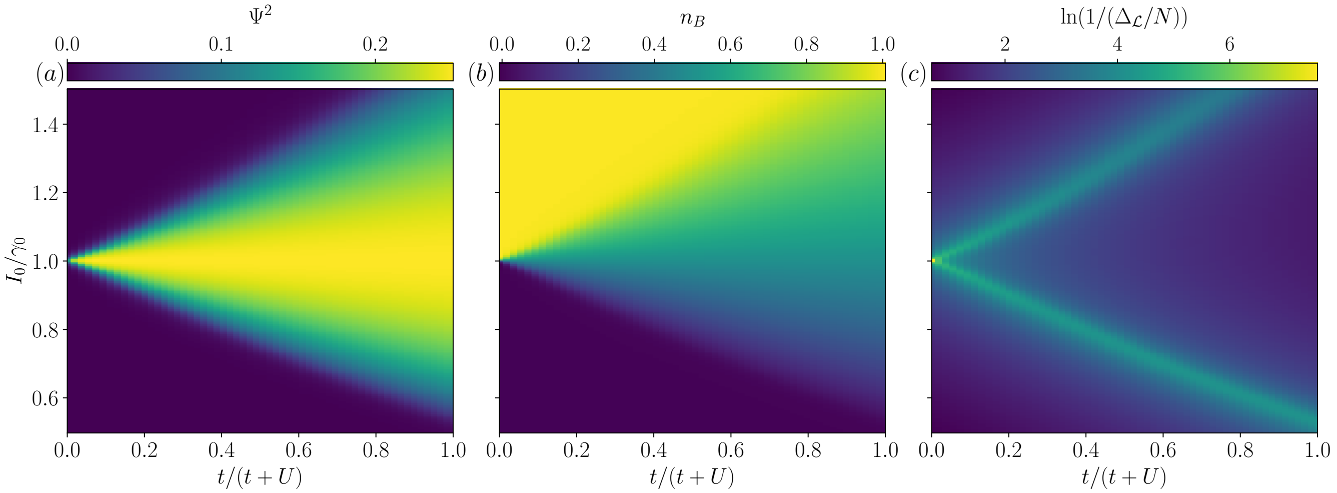

Fig. S7. We plot (a) Ψ^2, (b) n_B and (c) the inverse Liouvillian gap on a log scale, for $M = 1$. We see that the Liouvillian gap is smallest at phase boundaries.

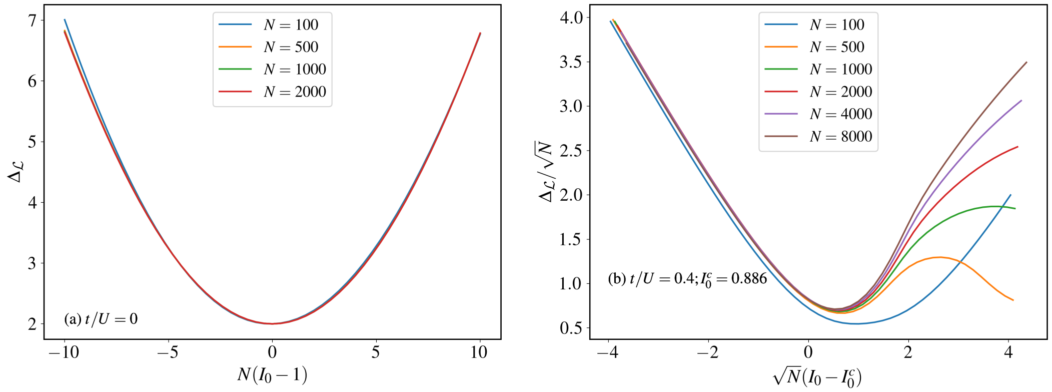

Fig. S8. We plot the Liouvillian gap scaling for the Mott-Mott (a) and Mott-superfluid (b) phase transitions for the $M = 1$ case. In both cases $\Delta_{\mathcal{L}} \sim N$ away from the critical point as in the superradiance case. The scaling collapse for the Mott-superfluid case has more finite-size effects due to the superfluid phase being an intermediate phase between two Mott phases.

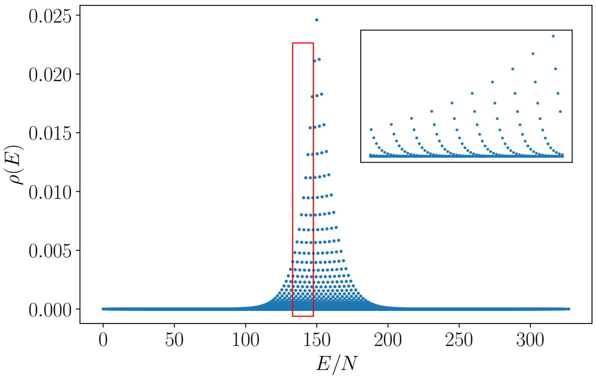

Fig. S9. The non-equilibrium steady state density matrix distribution for $M = 2$, at $I_0/\gamma_0 = 1.26, t/(t+U) = 0.1$. The inset is the zoomed-in region in the red rectangle.

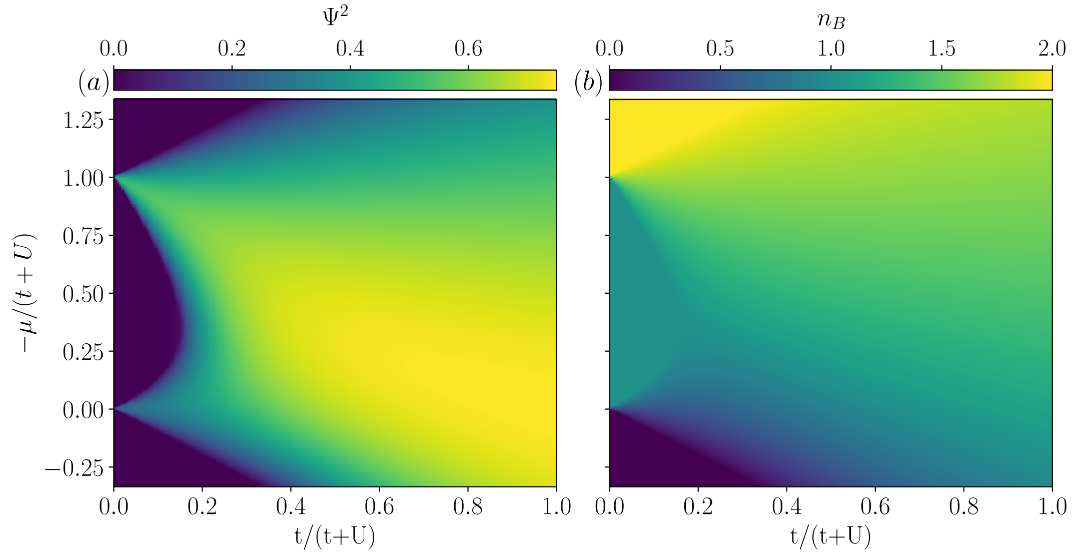

Fig. S10. The (zero-temperature) equilibrium phase diagrams for the all-to-all hopping Bose Hubbard model. The scaled observables Ψ^2 and n_B do not depend on N.

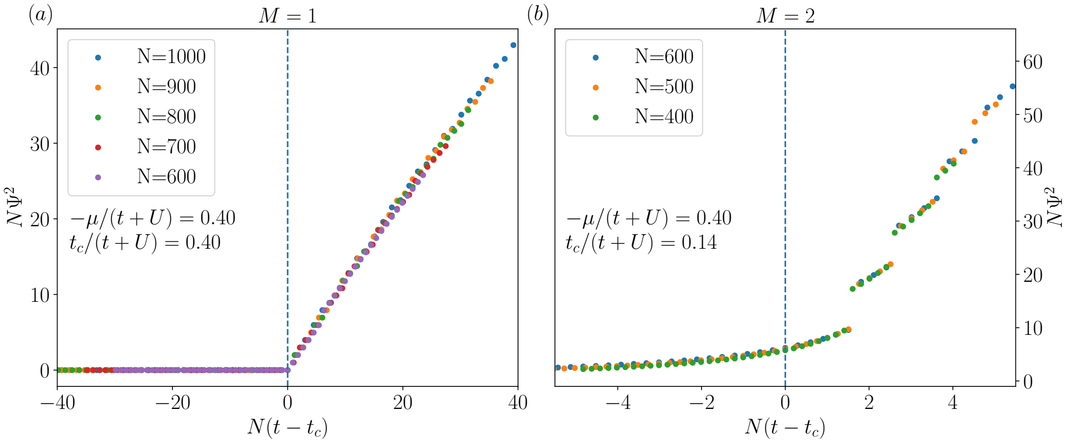

Fig. S11. We plot the scaling collapse for Ψ^2 for (a) $M = 1$ and (b) $M = 2$ respectively, for the transition from the Mott phase ($n_B = 1$) to the superfluid phase. We see that the equilibrium transition has $\lambda_s = 1$ as opposed to the nonequilibrium transition with $\lambda_s = 2$.

References

1. HP Breuer, F Petruccione, *The theory of open quantum systems.* (Oxford University Press, USA), (2002).
2. A Le Boité, G Orso, C Ciuti, Steady-state phases and tunneling-induced instabilities in the driven dissipative bose-hubbard model. *Phys. Rev. Lett.* **110**, 233601 (2013).
3. A Le Boité, G Orso, C Ciuti, Bose-hubbard model: Relation between driven-dissipative steady states and equilibrium quantum phases. *Phys. Rev. A* **90**, 063821 (2014).
4. R Hanai, PB Littlewood, Y Ohashi, Photoluminescence and gain/absorption spectra of a driven-dissipative electron-hole-photon condensate. *Phys. Rev. B* **97**, 245302 (2018).
5. S Sachdev, *Quantum Phase Transitions.* (Cambridge University Press), 2 edition, (2011).
6. Y Zhang, T Barthel, Criticality and phase classification for quadratic open quantum many-body systems. *Phys. Rev. Lett.* **129**, 120401 (2022).
7. MPA Fisher, PB Weichman, G Grinstein, DS Fisher, Boson localization and the superfluid-insulator transition. *Phys. Rev. B* **40**, 546–570 (1989).
